# Downregulation of HS6ST2 by miR-23b-3p enhances matrix degradation through p38 MAPK pathway in osteoarthritis

**DOI:** 10.1038/s41419-018-0729-0

**Published:** 2018-06-13

**Authors:** Yuanxu Guo, Zixin Min, Congshan Jiang, Wei Wang, Jidong Yan, Peng Xu, Ke Xu, Jing Xu, Mengyao Sun, Yitong Zhao, Safdar Hussain, Rui Zhang, Quancheng Wang, Yan Han, Fujun Zhang, Wenhua Zhu, Dongmin Li, Liesu Meng, Jian Sun, Shemin Lu

**Affiliations:** 1 Department of Biochemistry and Molecular Biology, School of Basic Medical Sciences, Xi’an Jiaotong University Health Science Center, 710061 Xi’an, China; 2 Department of Child Health Care, Shaanxi Provincial People’s Hospital, Shaanxi 710068 Xi’an, China; 3 Department of Human Anatomy, Histology and Embryology, School of Basic Medical Sciences, Xi’an Jiaotong University Health Science Center, 710068 Xi’an, China; 4 Xi’an Hong Hui Hospital, the Affiliated Hospital of Xi’an Jiaotong University Health Science Center, 710054 Xi’an, China; 5 Key Laboratory of Environment and Genes Related to Diseases (Xi’an Jiaotong University), Ministry of Education of China, 710061 Xi’an, China

## Abstract

Osteoarthritis (OA) is the most common form of arthritis involving major structural changes of peripheral joints and local or systemic inflammation and in lack of therapeutic approaches because of complexity of underlying molecular basis. Our previous work showed that HS6ST2, an enzyme involved in the transfer of sulfate, is downregulated in cartilage tissues of OA patients compared with normal donors, but little is known about its regulatory mechanism. In this study, we demonstrated that the expression of HS6ST2 was lower in OA-damaged cartilage than smooth cartilage from the same patient. In chondrocytes, HS6ST2 could be targeted by miR-23b-3p, which was higher expressed in OA-damaged cartilage. Under TNF-α stimulation, the expression of HS6ST2 was found inversely correlated with the expression of miR-23b-3p. Downregulation of HS6ST2 regulated by overexpression of miR-23b-3p and siRNAs against *HS6ST2* could enhance the protein level of MMP13 and aggravate the matrix degradation in chondrocytes. Increased expression of MMP13 depended on activity of p38 MAPK rather than total p38 MAPK level and was abrogated by HS6ST2 overexpression. Together, the results indicated that downregulated HS6ST2 targeted by miR-23b-3p promotes matrix degradation by activating p38 MAPK in chondrocytes and OA cartilage.

## Introduction

Osteoarthritis (OA) is a most common degenerative joint disorder and is characterized by degradation of articular cartilage, thickening of subchondral bone, and synovial inflammation^1,2^. OA may occur in response to inappropriate mechanical stress and local or systemic inflammation associated with genetic predisposition, obesity, and metabolic syndrome^3,4^. Although it has been considered as a “wear and tear” disease for many decades, recently the roles of pro-inflammatory factors, such as tumor necrosis factor-α (TNF-α), interleukin (IL)-1β, and IL-6, have been implicated in the OA pathogenesis. The inflammatory mediators produced by abnormal chondrocytes and synovial cells can trigger the cartilage extracellular matrix (ECM) degradation and break the cartilage homeostasis^5^. Enzymes responsible for degrading extracellular include two groups: matrix metalloproteinases (MMPs) and aggrecanases^6^. MMP13 belongs to the former group and is a major enzyme hydrolyzing type-II collagen, a dominant protein component in cartilage ECM^7,8^. Compared with other MMPs, MMP13 is expressed more strictly in connective tissue and is usually produced by only cartilage and bone during development^9,10^. Under stimulation of cytokines from mechanical injury and inflammation, MMP13 overexpression is regulated by complicated signals containing mitogen-activated protein kinase (MAPK) and nuclear factor (NF)-κB^11,12^. Emerging evidence from OA patients and rodent experimental arthritis supports that MMP13 plays a crucial role in the disease development and is believed as an important biomarker to reflect arthritis progress and therapeutic effects^13–15^.

Heparan sulfate proteoglycans are one kind of important ECM proteins covalently linked by polysaccharide side chains that are polymerized by enzymes and further modified by sulfation, deacetylation, and epimerization^16^. Sulfotransferases and sulfatases catalyze to add or release sulfate group to or from heparan sulfate residues, respectively^17,18^. The expression of heparan sulfate 6-O endosulfatases including Sulf1 and Sulf2 (Sulfs) are increased in human OA cartilage, and Sulf-deficient mice show much severe OA pathology^19,20^, indicating that enzymes involved in the sulfation of proteoglycan is associated with OA development.

Heparan sulfate 6-*O*-sulfotransferases (HS6ST) catalyze the transfer of sulfate group to C-6 (an exocyclic carbon) of the glucosamine residue in heparan sulfate. HS6STs belong to Golgi-resident enzymes and include three isoforms named HS6ST1, HS6ST2, and HS6ST3 in humans^21^. HS6STs are involved in tumorigenicity^21^, angiogenesis^22^, and neuron development^23^. In previous study, we found that HS6ST2 expression was significantly reduced in the cartilage of patients with OA and Kashin–Beck disease^24^. In addition, HS6ST2 might modulate fibroblast growth factor-2 signaling to affect chondrocyte growth and differentiation^25^. But the exact role of HS6ST2 is still elusive and the gene expression regulation of HS6ST2 especially in OA cartilage remains unknown.

MiRNAs are short non-coding RNAs with ~22 nucleotides that bind 3′-untranslated region (3′UTR) of mRNAs to regulate gene expression through either repressing translation or increasing mRNA degradation generally^26,27^. Recently, more studies have reported that miRNAs participate in chondrogenesis^28,29^, cartilage degradation, and OA development^30,31^. In previous study, we have identified miRNA repertoire in the different stages of articular development and also proved that miR-337 was associated with chondrogenesis through regulating TGFBR2 expression^32,33^. Considering the regulation of HS6ST2 expression in chondrocytes, we hypothesize that the downregulation of HS6ST2 could be mediated by a certain miRNA. Using miRNA target prediction algorithms, miR-23b-3p was assumed to bind mRNA sequence of *HS6ST2*. MiR-23b-3p is located in human chromosome 9q22.32 region with 900 base pairs. The region contains miR-23b/27b/24-1 cluster closed to encoded gene C9orf3^34^. Previous studies about miR-23b were focused on cancer^35,36^ and metabolic diseases^37,38^. But there is a study showing that miR-23b influences the differentiation of mesenchymal stem cell (MSC) into chondrocytes by directly targeting protein kinase A (PKA) mRNA to inhibit PKA signaling^39^.

In the present study, we first observed lower *HS6ST2* mRNA expression and higher expression of both miR-23b-3p and *MMP13* mRNA in the damaged cartilage compared with matched smooth cartilage from the same OA patients. Then we elucidated the roles of miR-23b-3p by directly targeting *HS6ST2* mRNA and intervened miR-23b-3p expression leading to downregulation of HS6ST2 in chondrocytes. The deregulation of HS6ST2 could result in increased expression of MMP13 by activating p38 MAPK pathway.

## Result

### There are relationships among the expression levels of HS6ST2, MMP13, and miR-23b-3p in damaged cartilage from OA patients

**Fig. 1:**
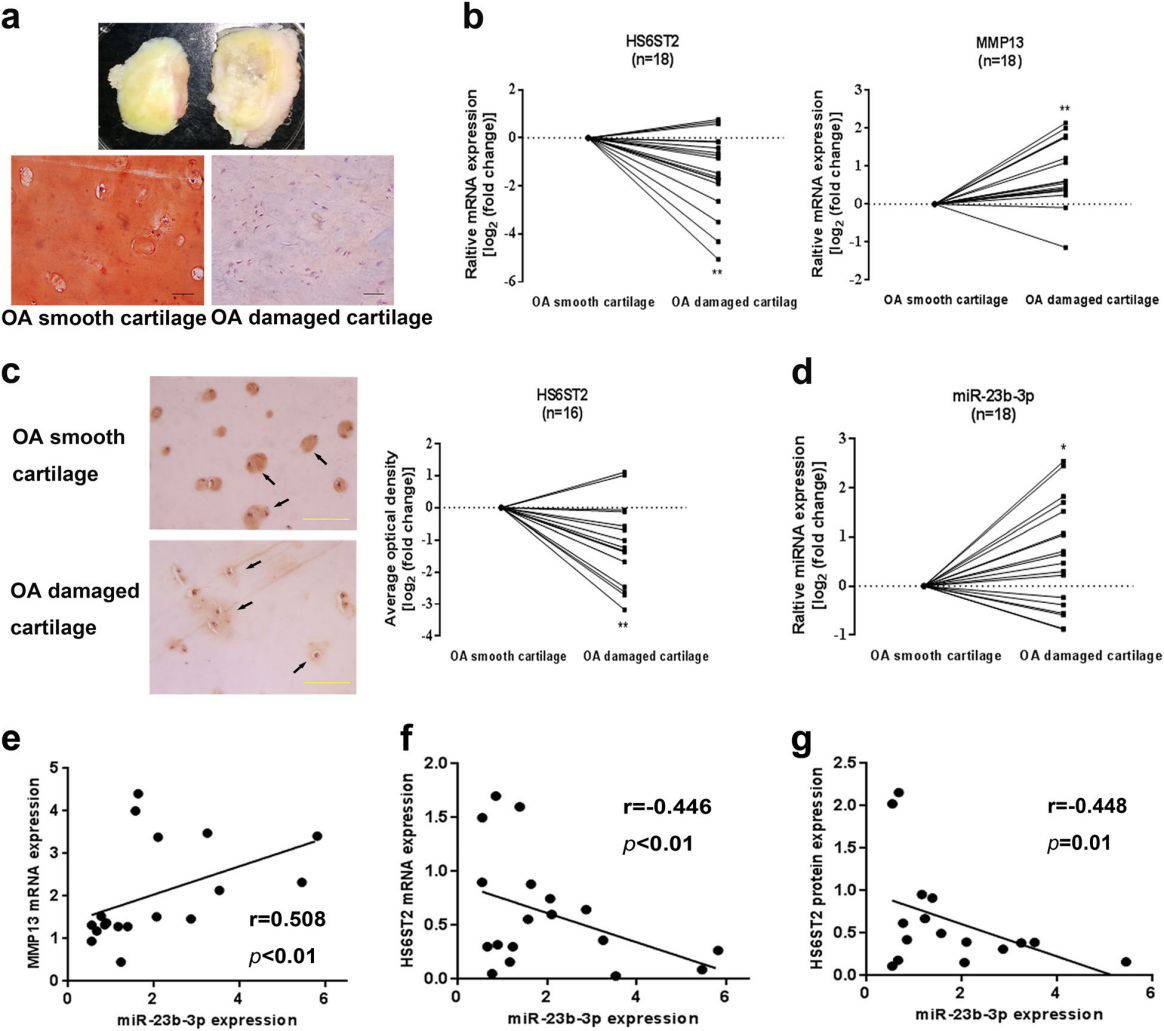
There are relationships among expressions of HS6ST2, MMP13, and miR-23b-3p in OA-damaged cartilage. **a** Representative macromorphological pictures of smooth cartilage (upper, left panel) and damaged cartilage (upper, right panel) from OA patients and representative Safranin O-fast green-stained histologic specimens of smooth OA cartilage and damaged OA cartilage. Scale bar: 50 μm. **b** Relative expression of *HS6ST2* mRNA (left panel) and *MMP13* mRNA (right panel) in damaged cartilage compared with smooth cartilage from the same OA patients (*n* = 18) were determined by RT-qPCR. **c** Left, representative immunohistochemical localization of HS6ST2 expression in OA smooth and damaged cartilage from same patient. Arrows stand for HS6ST2-positive cells. Right, statistical analysis of average optical density in immunohistochemical detection from OA patients (*n* = 16). Scale bar, 100 μm. **d** Relative expression of miR-23b-3p in damaged cartilage compared with smooth cartilage from the same OA patients (*n* = 18) were determined by RT-qPCR. **e** The correlation analysis between miR-23b-3p and *MMP13* mRMA expression from OA patients was performed. **f**, **g** The correlation analysis between miR-23b-3p and HS6ST2 expression from OA patients was performed. U6 snRNA was used as an internal control in RT-qPCR for miRNA and glyceraldehyde-3-phosphate dehydrogenase (GAPDH) was chosen for mRNA detection. Paired *t*-test was used to identify differences statistically between OA smooth cartilage group and OA-damaged cartilage group. *P < 0.05 and **P < 0.01. The correlation analysis was performed by using Spearman correlation

The OA-damaged cartilage and smooth cartilage from the same patients undergoing knee arthroplasty surgery were separated (Fig. 1a, upper panel) and validated by Safranin O-fast green staining (Fig. 1a, lower panel). *HS6ST2* mRNA expression (Fig. 1b, left panel) was attenuated and *MMP13* mRNA expression (Fig. 1b, right panel) was enhanced in damaged cartilage from 18 OA patients. Furthermore, we detected HS6ST2 protein in 16 pairs of OA patients using immunohistochemistry (IHC) and determined that HS6ST2 expression was significantly lower in OA-damaged cartilage tissues than matched smooth cartilage tissues (Fig. 1c and Supplementary Figure S1).

Bioinformatics results from TargetScan (www.targetscan.org), miRDB (www.mirdb.org), and miRanda (www.microrna.org) showed that miR-23b-3p is a candidate miRNA for targeting *HS6ST2* mRNA. The mature sequence of miR-23b-3p is conservative in humans, rats, and mice, and in the three species the 3′UTR of *HS6ST2* mRNA is matched with miR-23b-3p (Supplementary Figure S2). In 18 pairs of OA patients, it was found that the expression of miR-23b-3p was higher in OA-damaged cartilage tissues (Fig. 1d). The correlation analysis showed a positive correlation (*r* = 0.508, *P* < 0.01) between the miR-23b-3p and *MMP13* RNA level (Fig. 1e) and a negative correlation between miR-23b-3p and *HS6ST2* mRNA (*r* = −0.446, *P* < 0.01) or HS6ST2 protein (*r* = −0.448, *P* = 0.01) (Fig. 1f, g).

### *HS6ST2* mRNA is a target of miR-23b-3p

**Fig. 2:**
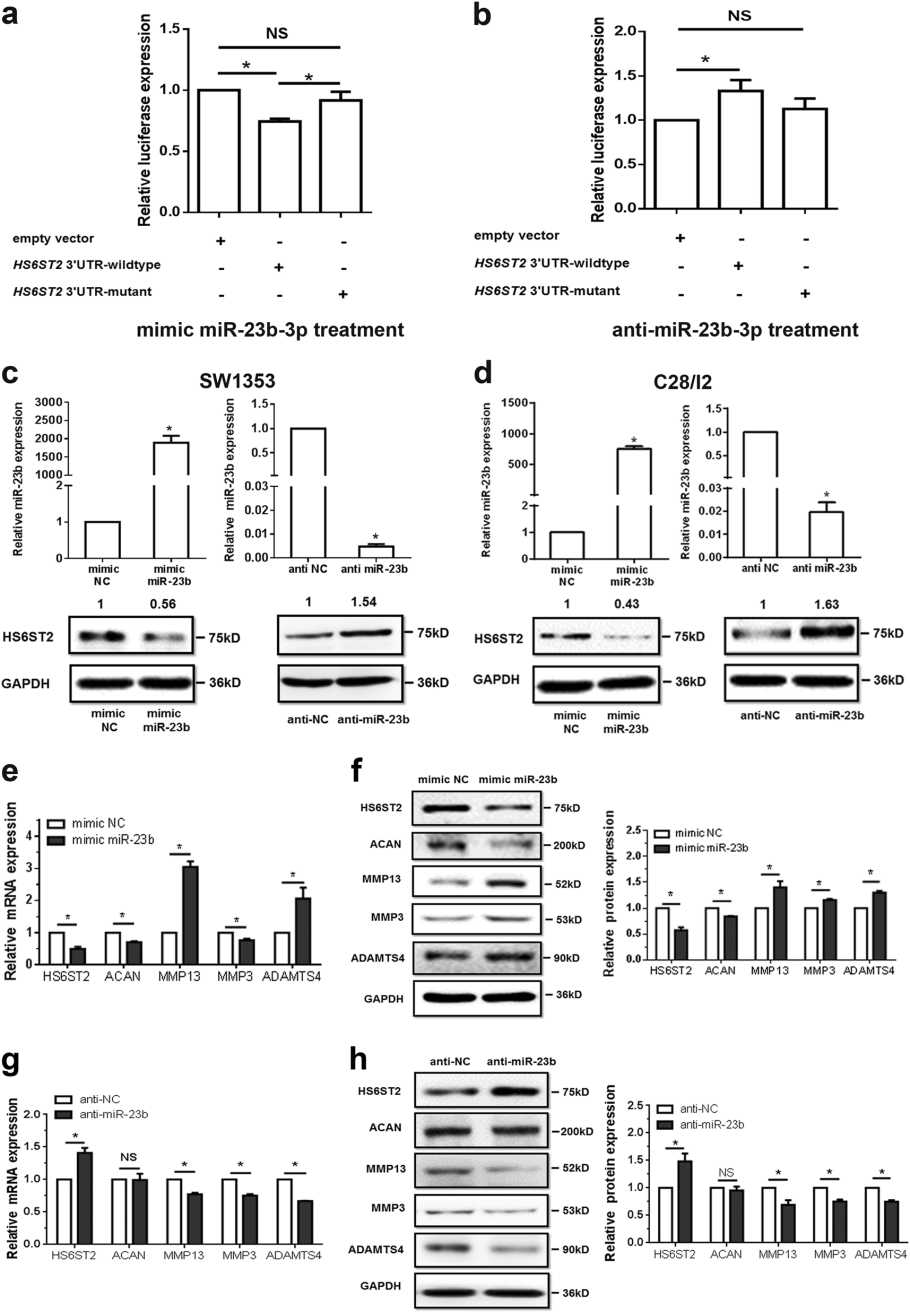
MiR-23b-3p inhibits HS6ST2 expression by targeting *HS6ST2* mRNA and effects specific gene expression in chondrocytes. **a**, **b** Dual luciferase reporter assay to validate target relationship between *HS6ST2* and miR-23b-3p. SW1353 cells were transfected with mimic miR-23b-3p (**a**) or anti-miR-23b-3p (**b**) by control vector, pmirGLO-*HS6ST2* wild-type 3′UTR vector or pmirGLO-*HS6ST2* mutant 3′UTR vector, respectively, for 48 h. **c**, **d** Stem-loop RT-qPCR and western blotting results in SW1353 cells (**c**) or C28/I2 cells (**d**) transfected with 10 nM mimic miR-23b-3p (left panel) or 50 nM anti-miR-23b-3p sequence (right panel). **e**, **f** RT-qPCR (**e**) and western blotting (**f**) results of cartilage-specific gene expression in SW1353 cells transfected with mimic miR-23b-3p or negative control. **g**, **h** RT-qPCR (**g**) and western blotting (**h**) results of cartilage-specific gene expression in SW1353 cells transfected with anti-miR-23b-3p or negative control. RNA was harvested at 24 h, while protein was isolated at 48 h after transfection. U6 snRNA was used as internal controls in miRNA stem-loop RT-qPCR detection and GAPDH was used as internal controls in mRNA RT-qPCR and western blotting detection. Bars represent standard error of the mean (SEM) from three independent experiments. Mann–Whitney *U* test was used to identify statistical differences between two groups. *P value < 0.05

To confirm whether *HS6ST2* mRNA is the target of miR-23b-3p, dual luciferase reporter assay was performed. SW1353 cells were transfected with both mimic miR-23b-3p and pmirGLO vectors, and a significant reduction was shown in pmirGLO-*HS6ST2* wild-type 3′UTR group relative to pmirGLO vector group but no significant reduction in pmirGLO-*HS6ST2* mutant 3′UTR group (Supplementary Figure S2c and Fig. 2a). The significant enhancement was demonstrated in the pmirGLO-*HS6ST2* wild-type 3′UTR group with anti-miR-23b-3p transfection rather than in the pmirGLO-*HS6ST2* mutant 3′UTR group (Fig. 2b), suggesting that miR-23b-3p could bind to the 3′UTR of human *HS6ST2* mRNA.

The expression of miR-23b-3p significantly increased under transfection of mimic miR-23b-3p (as much as 1500 times) compared with mimic negative control (NC) in SW1353 cells (Fig. 2c, left panel), and correspondingly the transfection of anti-miR-23b-3p sequences could suppress the level of miR-23b-3p dramatically (over 99%) (Fig. 2c, right panel). The protein expression of HS6ST2 was downregulated after the administration of mimic miR-23b-3p, whereas transfection with the anti-miR-23b-3p sequences could upregulate the protein expression of HS6ST2 (Fig. 2c, lower panel). The results were confirmed in another human chondrocyte cell line C28/I2^40^ (Fig. 2d). Collectively, the above-mentioned results suggested that miR-23b-3p could regulate HS6ST2 protein expression in chondrocytes by targeting *HS6ST2* mRNA 3′UTR.

### Intervention of miR-23b-3p affects specific gene expression in chondrocytes

To further confirm the roles of miR-23b-3p in chondrocytes, the mimic of miR-23b-3p was used to alter the expression of miR-23b-3p. MMP13 and ADAMTS4 significantly increased under treatment of mimic miR-23b-3p (Fig. 2e, f). The downregulation of HS6ST2 upon miR-23b-3p overexpression was observed and Aggrecan Core Protein or Cartilage-Specific Proteoglycan Core Protein (ACAN) also decreased at both protein and mRNA levels under overexpression of miR-23b-3p (Fig. 2e, f). Transfection with anti-miR-23b-3p led to significant downregulation of MMP13, MMP3, and ADAMTS4 in chondrocytes and upregulation of HS6ST2 (Fig. 2g, h). The findings provided evidence that miR-23b-3p could affect ECM metabolism in human chondrocytes.

### TNF-α-treated chondrocytes increase matrix degradation and decreases HS6ST2 expression regulated by miR-23b-3p

**Fig. 3:**
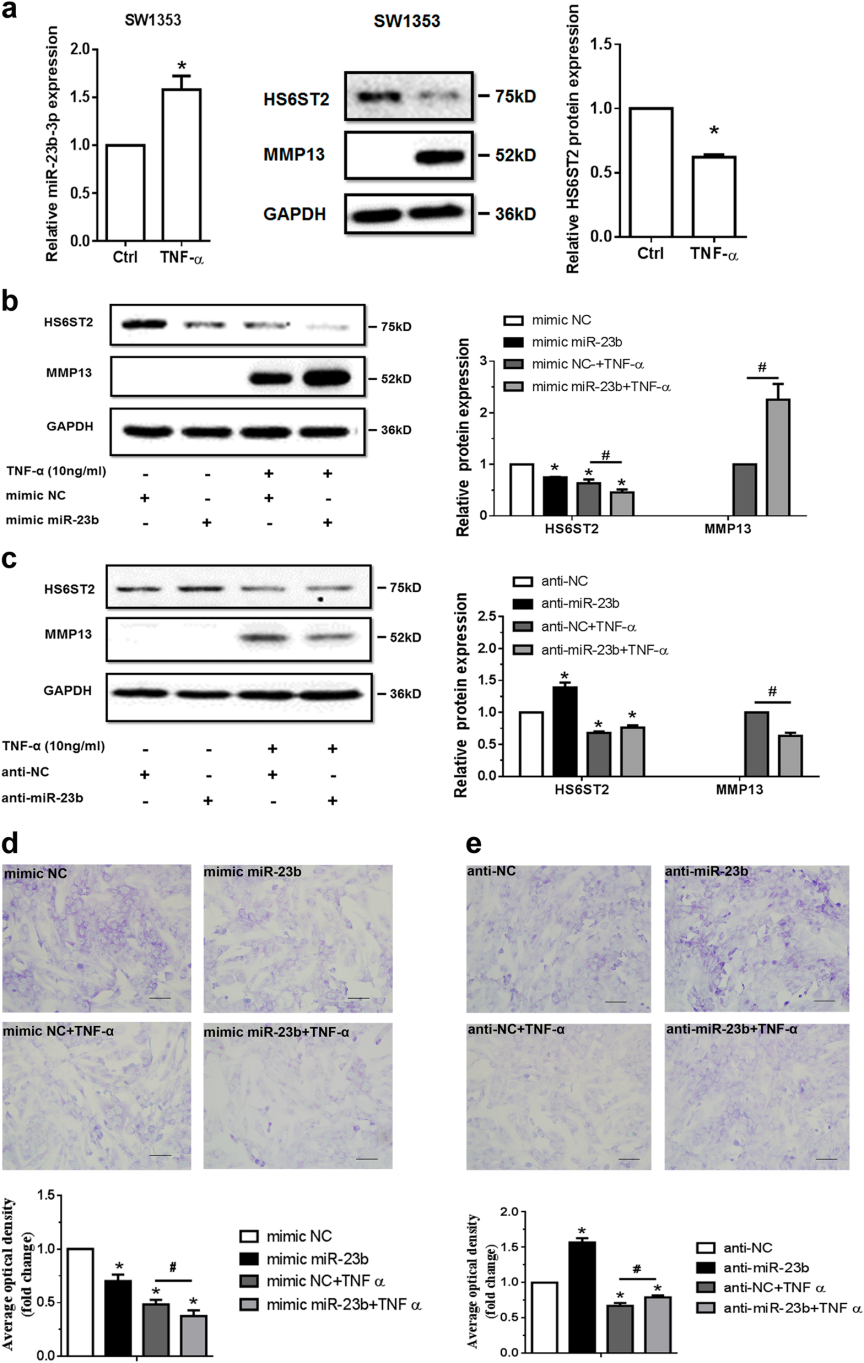
TNF-α-treated chondrocytes increases matrix degradation regulated by miR-23b-3p. **a** Stem-loop RT-qPCR result of miR-23b-3p (left panel) and protein levels of HS6ST2 and MMP13 (right panel) were assayed in SW1353 cells under stimulation by 10 ng/ml TNF-α for 24 h. **b**, **c** Western blotting result of HS6ST2 and MMP13 protein in SW1353 cells transfected with 10 nM mimic miR-23b-3p (**b**) or 50 nM anti-miR-23b-3p sequence (**c**) and stimulated by 10 ng/ml TNF-α for 24 h. **d**, **e** Toluidine blue staining results of SW1353 cells transfected with 10 nM mimic miR-23b-3p (**d**) or 50 nM anti-miR-23b-3p sequence (**e**) and stimulated by 10 ng/ml TNF-α for 24 h. Scale bar, 100 μm. Lower panel, statistical analysis of average optical density of matrix staining of toluidine blue. Asterisk (*): compared with mimic NC or anti-NC group. U6 snRNA and GAPDH were used as internal controls in RT-qPCR for miRNA and western blotting detection, respectively. Bars represent standard error of the mean (SEM) from three independent experiments. One representative result and quantitative data from three independent western blotting and toluidine blue staining. Mann–Whitney *U* test was used to identify statistical differences between two groups. Asterisk (*) or hash (^#^) stands for *P* value <0.05

TNF-α was selected to stimulate SW1353 cells and C28/I2 cells. As shown in Fig. 3a and Supplementary Figure S3, miR-23b-3p level increased under TNF-α treatment; meanwhile, the protein level of HS6ST2 demonstrated downregulation in both SW1353 cells and C28/I2 cells. To determine whether miR-23b-3p could influence matrix degradation induced by TNF-α, SW1353 cells were transfected with mimic miR-23b-3p under TNF-α stimulation, and the MMP13 expression was enhanced more severely under the stimulation of both miR-23b-3p and TNF-α compared with TNF-α alone (Fig. 3b), and anti-miR-23b-3p could inhibit the MMP13 expression induced by TNF-α (Fig. 3c). The matrix content of chondrocytes was determined by toluidine blue staining at 48 h after transfection. After image analysis, the mean optical density of the staining under miR-23b-3p transfection was significantly lower than that of the NC group with or without TNF-α (Fig. 3d), and the matrix staining was enhanced by anti-miR-23b-3p transfection (Fig. 3e), suggesting that miR-23b-3p might take part in the catabolism pathway induced by TNF-α.

**Fig. 4:**
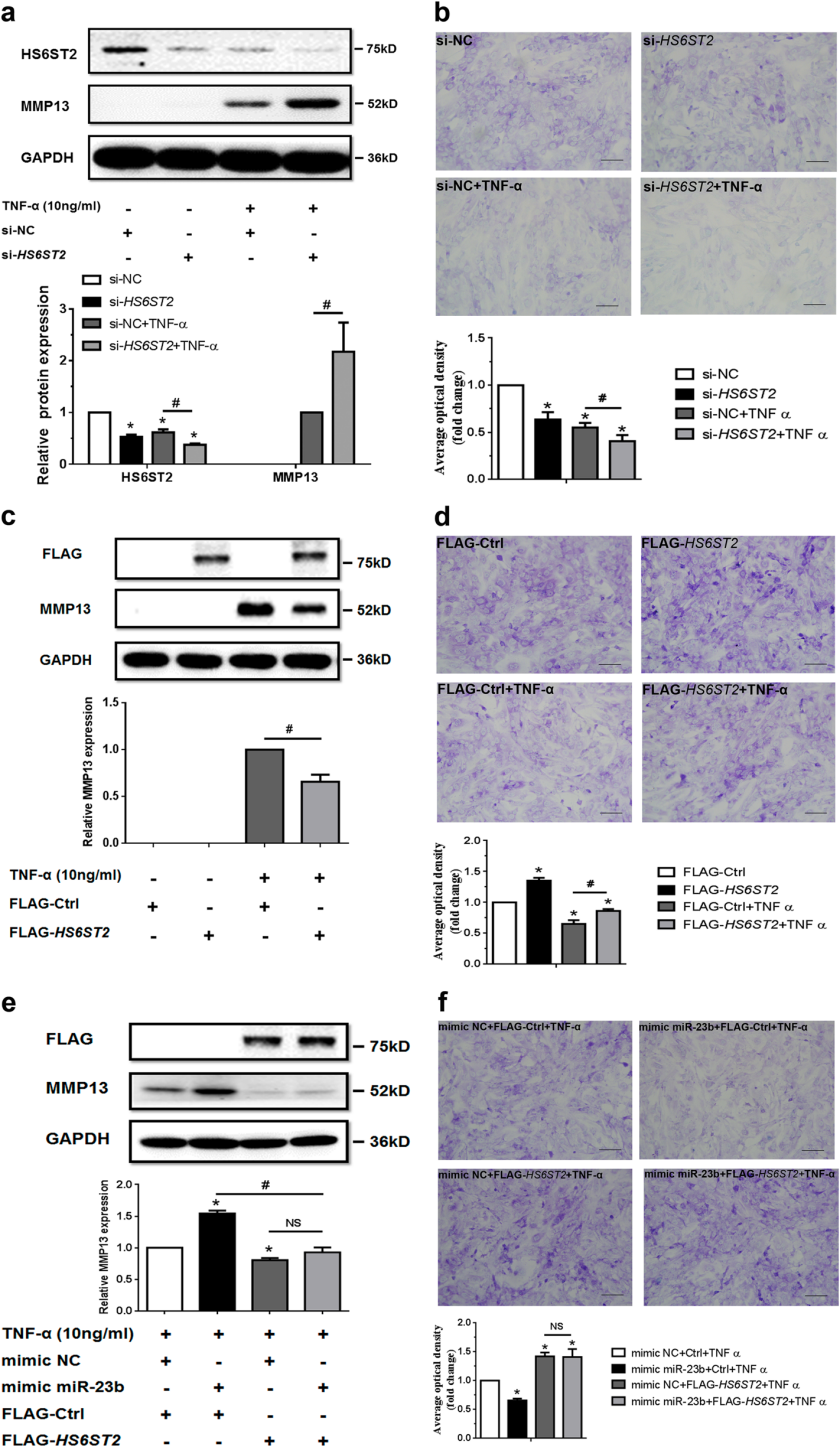
MiR-23b-3p could enhance matrix degradation in human chondrocytes via regulating HS6ST2. **a**, **b** SW1353 cells transfected with 50 nM *HS6ST2* siRNA mixture (containing three target sequences, si-*HS6ST2*) or negative control (si-NC) were stimulated by 10 ng/ml TNF-α for 24 h. Protein level of HS6ST2 and MMP13 were assayed by western blotting (**a**) and matrix content of chondrocytes was determined by toluidine blue staining (**b**). Asterisk (*): compared with the si-NC group. **c**, **d** SW1353 cells transfected with empty vector (FLAG-Ctrl) or pcDNA3.1-FLAG-*HS6ST2* vector (FLAG-*HS6ST2*) for 24 h were stimulated by TNF-α for another 24 h. The protein expression of MMP13 was assayed by western blotting (**c**) and matrix content of chondrocytes was determined by toluidine blue staining (**d**). Asterisk (*): compared with the FLAG-Ctrl group. **e**, **f** Under stimulation with TNF-α, SW1353 cells were treated with mimic NC or mimic miR-23b-3p for 24 h and then transfected with empty vector (FLAG-Ctrl) or pcDNA3.1-FLAG-*HS6ST2* vector (FLAG-*HS6ST2*) for another 24 h to observe the rescuing effect of mimic miR-23b-3p in MMP13 expression (**e**) and matrix content (**f**). Asterisk (*): compared with the mimic NC group. GAPDH was used as internal controls in western blotting detection. In toluidine blue staining results, scale bar, 100 μm. Lower panel, statistical analysis of average optical density of matrix staining of toluidine blue. Bars represent standard error of the mean (SEM) from three independent experiments. Mann–Whitney *U* test was used to identify statistical differences between two groups. Asterisk (*) or hash (^#^) stands for *P* value <0.05. NS stands for not significant

The specific small interfering RNA (siRNA) against *HS6ST2* (si-*HS6ST2*) was used to intervene HS6ST2 expression. Knockdown of HS6ST2 dramatically enhanced the MMP13 expression induced by TNF-α and attenuated the matrix staining with or without TNF-α similar to the effect of mimic miR-23b-3p (Fig. 4a, b). In C28/I2 cells, upregulation of MMP13 from ectopic miR-23b-3p or si-*HS6ST2* was also shown under TNF-α stimulation (Supplementary Figure S4).

To confirm the matrix degradation caused by mimic miR-23b-3p via the target relationship between miR-23b-3p and *HS6ST2* mRNA, the plasmid for ectopic expression of HS6ST2 with FLAG tag was constructed. MMP13 protein level decreased and matrix staining increased under overexpression of HS6ST2 treated with TNF-α (Fig. 4c, d). Although the protein level of MMP13 could be upregulated by miR-23b-3p, the upregulation of MMP13 from ectopic miR-23b-3p was attenuated by overexpression of HS6ST2 (Fig. 4e). Under TNF-α stimulation, the matrix staining was significantly reduced with ectopic miR-23b-3p, but the degradation was rescued by HS6ST2 overexpression (Fig. 4f), suggesting that miR-23b-3p participates in matrix degradation via regulating HS6ST2 expression.

### Phosphorylation of p38 MAPK is dependent on HS6ST2 mediated by miR-23b-3p

**Fig. 5:**
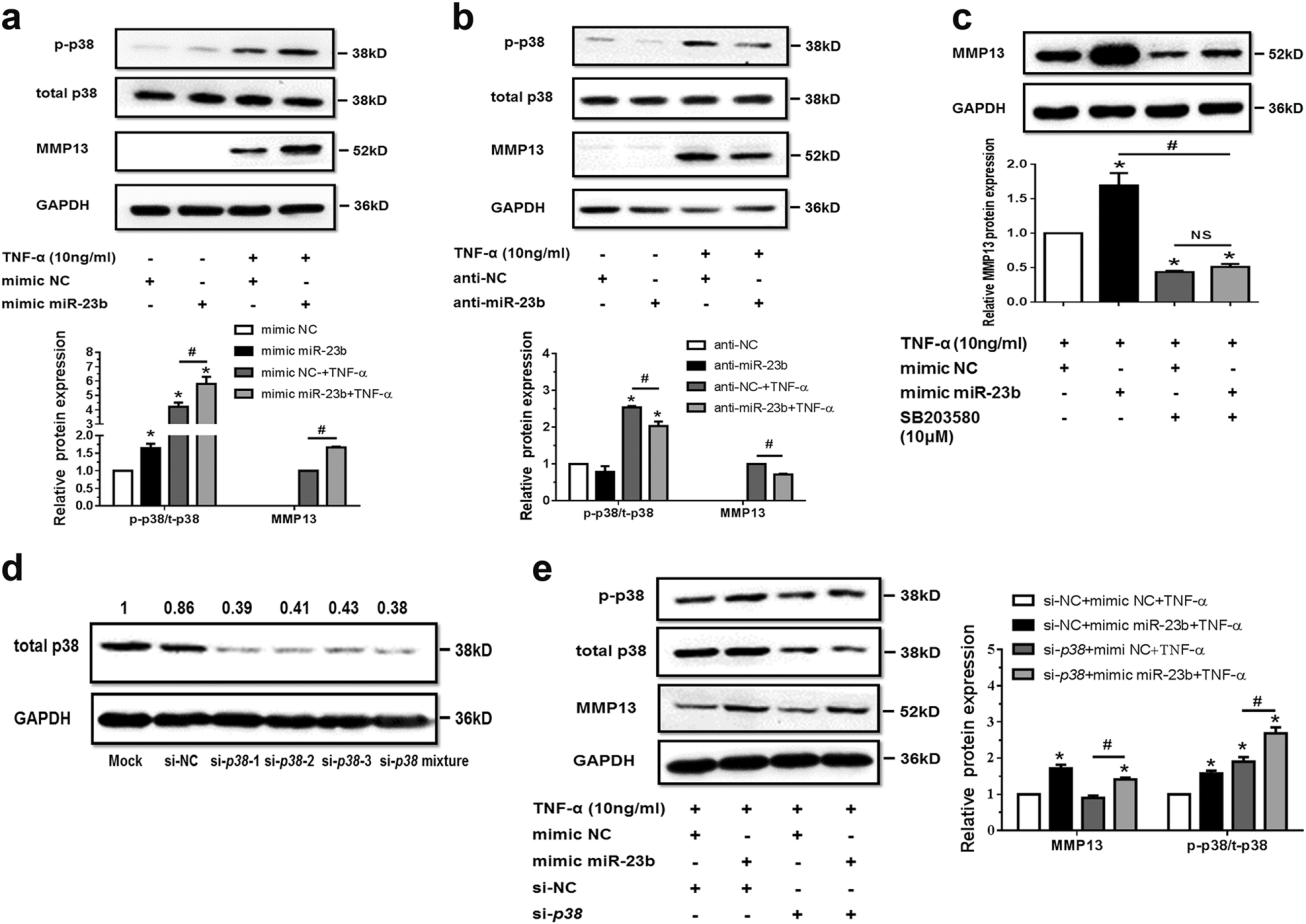
MiR-23b-3p could enhance matrix degradation by means of regulating activity of p38 MAPK in human chondrocytes under TNF-α treatment. **a**, **b** SW1353 cells were transfected by mimic miR-23b-3p (**a**) or anti-miR-23b-3p sequence (**b**) with or without TNF-α for 24 h, and the protein expression of phosphorylation form of p38 MAPK (p-p38), total p38 MAPK, and MMP13 was detected by western blotting. Asterisk (*): compared with the mimic NC (**a**) or anti-NC group (**b**). **c** SW1353 cells were treated with mimic miR-23b-3p with or without p38 MAPK inhibitor SB203580 (10 μM) under stimulation of TNF-α. The protein expression of MMP13 was determined by western blotting. Asterisk (*): compared with the mimic NC group. **d** Interfering efficiency of siRNAs against *p38 MAPK* was determined by western blotting under 50 nM p38 siRNA, 50 nM negative control (NC), and Mock (transfection regent only) transfection for 48 h. **e** Under stimulation with TNF-α, SW1353 cells were transfected by mimic miR-23b-3p under treatment with si-*p38* mixture (containing three siRNA target sequences), and p-p38, total p38, and MMP13 were determined by western blotting. Asterisk (*): compared with mimic NC or si-NC. Each relative expression of phosphorylation form was normalized by the total form. GAPDH was used as internal controls in western blotting detection. Mann–Whitney *U* test was used to identify statistical differences between two groups. Asterisk (*) or hash (^#^) stands for *P* value <0.05. NS stands for not significant

p38 MAPK, c-Jun N-terminal kinase (JNK) MAPK, and NF-κB signaling, regulated by extracellular stress factors, are crucial downstream pathways of TNF-α during inflammation and matrix degradation^5^. Our data showed that, with or without TNF-α, p38 MAPK pathway was significantly regulated during gain or loss of miR-23b-3p function rather than JNK MAPK and NF-κB signaling (Supplementary Figure S5). The protein expression of MMP13 and p-p38 were both positively regulated by miR-23b-3p (Fig. 5a, b), suggesting that p38 MAPK signaling might participate in upregulation of matrix degradation from ectopic miR-23b-3p.

The p38 MAPK inhibitor SB203580 was used in chondrocytes transfected with miR-23b-3p under stimulation with TNF-α. The expression of MMP13 induced by TNF-α could be inhibited by SB203580 (Supplementary Figure S6a), and upregulation of MMP13 induced by miR-23b-3p could also be attenuated after treatment of SB203580 in SW1353 cells (Fig. 5c and Supplementary Figure S6b) and C28/I2 cells (Supplementary Figure S7a), indicating that miR-23b-3p could regulate the expression of MMP13 depending on p38 MAPK. Furthermore, after treatment of si-*p3*8 MAPK, the level of phosphorylation form of p38 MAPK and upregulation of MMP13 via miR-23b-3p were not attenuated (Fig. 5d, e), although total p38 MAPK was downregulated, suggesting that upregulation of MMP13 via miR-23b-3p could be influenced by the activity of p38 MAPK (phosphorylation form of p38 MAPK) rather than of total p38 MAPK.

**Fig. 6:**
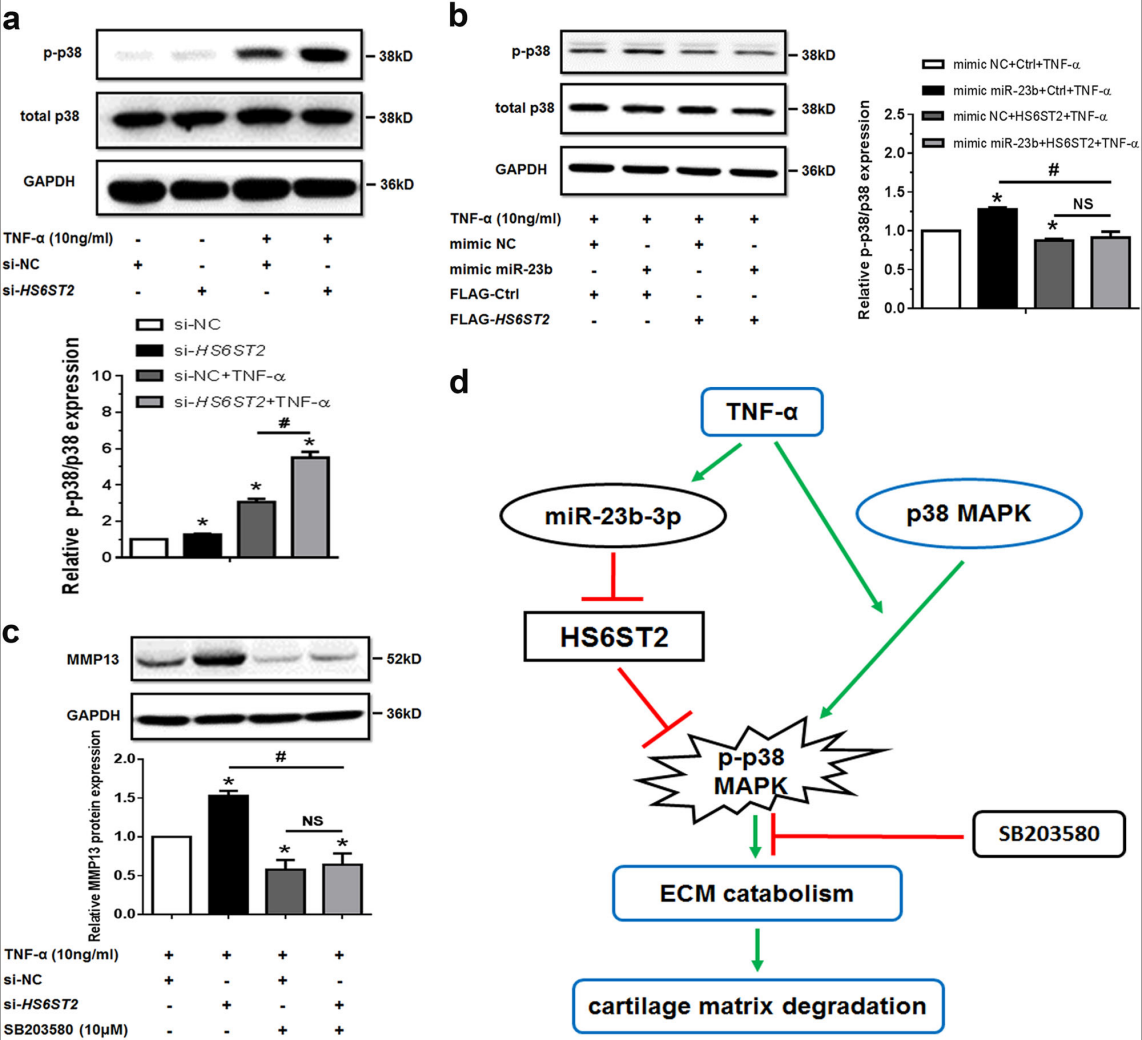
HS6ST2 could regulate the matrix degradation depending on the activity of p38 MAPK. **a** SW1353 cells were transfected by si*-HS6ST2* or negative control (si-NC) with or without TNF-α for 24 h, and the protein expression of p-p38 and p38 was detected by western blotting. Asterisk (*): compared with the si-NC group. **b** Under stimulation with TNF-α, SW1353 cells were transfected by empty vector (FLAG-Ctrl) or pcDNA3.1-FLAG-*HS6ST2* vector (FLAG-*HS6ST2*) under treatment with mimic miR-23b-3p, and p-p38 and total p38 were determined by western blotting. Asterisk (*): compared with the mimic NC group. **c** SW1353 cells were transfected with *HS6ST2* siRNA with or without p38 MAPK inhibitor SB203580 (10 μM) after treatment of TNF-α, and the protein expression of MMP13 was determined by western blotting. Each relative expression of phosphorylation form was normalized by the total form. **d** Schematic representation of miR-23b-3p–HS6ST2 axis-mediated catabolic effects in human chondrocyte. GAPDH was used as internal controls in western blotting detection. Mann–Whitney *U* test was used to identify statistical differences between two groups. Asterisk (*) or hash (^#^) stands for *P* value <0.05. NS stands for not significant

Under the treatment of si-*HS6ST2*, p-p38 significantly increased (Fig. 6a) with treatment of TNF-α and HS6ST2 overexpression could also attenuate the upregulation of p-p38 from ectopic miR-23b-3p (Fig. 6b). The results from C28/I2 cells also showed that phosphorylation of p38 MAPK could be influenced by ectopic miR-23b-3p or si-*HS6ST2* (Supplementary Figure S4). Additionally, upregulation of MMP13 under si-*HS6ST2* transfection was also blocked by SB203580 (Fig. 6c, Supplementary Figure S6c and Supplementary Figure S7b). Above findings provided evidence that HS6ST2 mediated by miR- 23b-3p might regulate cartilage degradation depending on the activity of p38 MAPK.

## Discussion

The present study provided evidence to support a novel critical homeostatic mechanism that HS6ST2 as a key regulator in cartilage degradation is mediated by miR-23b-3p in cartilage. Previous reports showed that HS6ST2 expression was significantly reduced in the cartilage of OA patients and the change of HS6ST2 could alter the expression of cartilage-related genes^24^, suggesting a pivotal role of HS6ST2 during chondrocyte differentiation and progression of OA. However, the molecular mechanisms for HS6ST2-induced cartilage-specific gene expression and possible regulators for HS6ST2 expression remain unclear.

Hence, in this study we first assessed the HS6ST2 expression levels in OA-damaged cartilage tissues and the matched smooth cartilage tissues. In OA-damaged cartilage tissues, HS6ST2 was reduced at RNA and protein levels, while *MMP13* mRNA was enhanced, which are consistent with published reports about overexpression of MMP13 in OA-damaged cartilage^41^ and our previous study^24^.

Second, we examined the posttranscriptional regulation of HS6ST2 expression by miRNAs in chondrocytes and assumed that its expression could be regulated by miR-23b-3p. Bioinformatics results showed the conservation of target relationship between *HS6ST2* mRNA and miR-23b-3p in humans, mice, and rats (Supplementary Figure S2). There are other candidate miRNAs also targeting HS6ST2. miR-140, a well-known cartilage-specific miRNA, was predicted to bind 3′UTR of *HS6ST2* mRNA, for example. There have been several reports for miR-140 verifying its targeting gene, expression regulation, and roles in cartilage development and arthritis progression^42–44^. miR-23b-3p was selected for further study as a regulator of HS6ST2 expression rather than other candidate miRNAs based on the consistent results from several prediction algorithms, conservation in various species, and innovation in cartilage biology.

From OA patients, the increasing of miR-23b-3p in OA-damaged cartilage tissues related to matched smooth cartilage tissues was determined, which is in accord with previous large-scale microarray miRNA analysis^45^. Furthermore, the positive relationship between miR-23b-3p and MMP13 had been observed in the present study, indicating that overexpression of miR-23b-3p is an important factor in cartilage matrix degradation and OA pathogenesis. Dual-luciferase assay and protein detection after transfection indicated that miR-23b-3p could bind to the 3′UTR of human *HS6ST2* mRNA and regulates its expression in chondrocytes. The observation that HS6ST2 in damaged cartilage tissue manifested lower expression and was downregulated by miR-23b-3p in chondrocytes implicated that relationship between HS6ST2 and miR-23b-3p might be an important regulatory mechanism in OA progression.

Pro-inflammatory cytokines, such as TNF-α and IL-1β, play crucial roles in OA pathogenesis, which could promote catabolism in arthritic cartilage and enhance the expression of matrix-degradation genes^5^. In present study, TNF-α was selected in inducing matrix degradation of chondrocyte. Under treatment of TNF-α, the protein level of HS6ST2 was reduced, while the miR-23b-3p level was elevated, suggesting that the downregulation of HS6ST2 was mediated probably by upregulating miR-23b-3p in catabolism of chondrocyte. NF-κB pathway known to play an important role in the pathogenesis of OA^5^ has been proved as the upstream regulation of miR-23b-3p expression, which was reported in the metabolism and cancer development^37,46^. Hence, increasing of miR-23b-3p under TNF-α stimulation in our study might depend on the activation of NF-κB signal pathway.

MiR-23b has been found to induce chondrogenic differentiation in the differentiation of MSC into chondrocyte^39^. In this study, we intervened the expression of miR-23b-3p in mature cartilage cells with miR-23b-3p mimic or anti-miR-23b-3p, and we found that MMP13 expression and matrix content were affected by the change of miR-23b-3p in chondrocytes under TNF-α treatment. Even without TNF-α, both mimic miR-23b-3p and anti-miR-23b-3p could still influence the expression of cartilage-related catabolic genes and matrix content of chondrocytes. Together, our data support that miR-23b-3p could affect the catabolism of mature chondrocyte besides its role in the differentiation of MSC into chondrocytes.

In chondrocytes, downregulation of HS6ST2 could enhance the expression of MMP13 and aggravate matrix degradation similar to ectopic miR-23b-3p and HS6ST2 overexpression could rescue the detrimental effect of miR-23b-3p. Exogenous HS6ST2 with FLAG tag could improve the decline of HS6ST2 from miR-23b-3p and attenuate the expression of MMP13 and loss of matrix content in the activated phase of TNF-α treatment, and FLAG-HS6ST2 protein was not influenced by miR-23b-3p because of 3′UTR deleting. Thus we have confirmed that miR-23b-3p participates in matrix degradation via regulating HS6ST2 expression.

In order to verify the mechanism of MMP13 upregulation by HS6ST2 as well as miR-23b-3p, the downstream signal pathways of TNF-α including NF-κB, p38 MAPK and JNK MAPK^5^ were detected. Activation of p38 MAPK pathway was altered by change of miR-23b-3p or HS6ST2, which is consistent with previous reports that activity of p38 MAPK signaling pathway induces ECM degradation^47–49^. Catalytic inhibitors of p38 MAPK SB203580 could block p38 MAP kinase activity^50^, and the expression of MMP13 induced by TNF-α could be attenuated by SB203580 but more seriously under miR-23b-3p or si-*HS6ST2* treatment. Under treatment of si-*p38*, the phosphorylation of p38 MAPK was not attenuated dramatically though the total p38 MAPK was downregulated seriously, and increasing of MMP13 and p-p38 from ectopic miR-23b-3p could not be blocked, which might belong to feedback regulation. Above results suggested that enhancement of MMP13 regulated by miR-23b-3p and downregulation of HS6ST2 might depend on activity of p38 MAPK rather than total p38 MAPK.

Our results suggested that there was a close relationship between HS6ST2 and activation of p38 MAPK, and we assumed that HS6ST2 could interact with some proteins that influenced activity of p38 MAPK in posttranscriptional regulatory mechanism. We checked MEK3/6 (specific activators for p38 MAPK)^51^ and DUSP10 (dual-specificity protein phosphatases 10) that dephosphorylates the stress-activated p38 MAPK^52,53^ by co-immunoprecipitation in our system. But the results showed no interaction between HS6ST2 and above-mentioned proteins (Supplementary Figure S8). The exact mechanism of relationship between HS6ST2 and activation of p38 MAPK needs further exploring.

In conclusion, we found that, compared with smooth cartilage, the expression of HS6ST2 is lower where miR-23b-3p and *MMP13* mRNA are higher in damaged cartilage in OA patients, and miR-23b-3p binds with 3′UTR of *HS6ST2* mRNA and inhibit its expression in human chondrocytes. HS6ST2 regulated by miR-23b-3p affects MMP13 expression via enhancing the phosphorylation of p38 MAPK rather than total p38 MAPK change (see Fig. 6d). The work is the first to demonstrate the regulation of HS6ST2 by a miRNA during cartilage matrix metabolism, and HS6ST2 might play a pivotal role in matrix degradation. Further studies such as administrating miR-23b-3p mimic/inhibitors (gain/loss function of its target HS6ST2, and other possible targets) on mouse OA models are needed to provide more solid evidence in future. The findings not only provide new insights into the role of sulfotransferase in matrix degradation regulation but also raise possibilities to intervene HS6ST2 as well as miR-23b-3p for developing a novel therapeutic strategy of OA.

## Materials and Methods

### Patients and articular cartilage collection

OA patients were diagnosed according to the modified Outerbridge classification by Xi’an Honghui Hospital, Xian Jiaotong University. Articular cartilage samples were collected from 18 patients from Shaanxi province, China with knee OA undergoing knee arthroplasty surgery (14 women and 4 men; age (mean ± SEM) 66.6 ± 4.2 years). After washing with sterile phosphate-buffered saline (PBS) buffer, portions of cartilage with smooth articular surface and portions with damaged articular surface were used for histologic stain, RNA extraction, and IHC. This study was performed with the approval of the Ethical Committee of the Xi’an Jiaotong University Health Science Center, and all individuals provided full written informed consent before the operative procedure.

### RNA extraction and quantitation analysis in cartilage specimens

For RNA extraction, cartilage tissues (*n* = 18) from smooth and damaged articular surface of the same patients were harvested and divided into small pieces (<2 × 2 mm^2^). The pieces were frozen in liquid nitrogen immediately. Total RNA was isolated with Trizol reagent (Thermo Fisher, catalog 15596-026) according to the manufacturer’s protocol. cDNA was synthesized from 2 μg of total RNA in 20 μl of reaction system using the Transcriptor cDNA Synthesize Kit (Roche, catalog 04897030001) with OligdT primer for detecting mRNA expression. Meanwhile, the same cDNA sample was synthesized from 1 μg of total RNA with the mir-X miRNA First-Strand Synthesis Kits (Clontech, catalog 638315). Gene expression levels were detected by quantitative PCR (qPCR) with the SYBR Green System (Roche, catalog 04913850001). The primers for measuring mRNA are shown in Supplementary Table S2. The forward primer for miR-23b-3p detection was designed by Tiangen Biotech (Beijing, China) and reverse primer for miRNA measure was from the mir-X miRNA First-Strand Synthesis Kits. Quantification of the relative expression levels was determined by ΔΔCt method.

### Histology and IHC

Human cartilage tissues (*n* = 16) were fixed in 4% buffered paraformaldehyde for at least 48 h and subsequently decalcified with buffered EDTA (12.5% EDTA, pH 7.4). Dehydrated samples were embedded in paraffin, and 5 μm-thick sections were cut. Tissue sections were deparaffinized in xylenes and rehydrated through a graded series of alcohols. Representative sections from smooth cartilage and damaged cartilage of OA patients were stained with Safranin O and counterstained with fast green. For IHC, after 3% H_2_O_2_ treatment and antigen retrieval, all tissue sections were blocked at room temperature with 5% bovine serum albumin (BSA) for 30 min and then incubated with primary antibody at the dilution stated: anti-HS6ST2 (1:50, Santa Cruz, catalog sc-98287). After sequential incubations with biotinylated secondary antibody (BOSTER, catalog SA1022, China) and horseradish peroxidase-conjugated avidin (BOSTER, catalog SA1022, China), protein staining was performed using the Diaminobenzidine Substrate Kit (BOSTER, catalog SA2022, China). Samples were counterstained with hematoxylin, and brown staining indicated the HS6ST2 immunoreactivity. The results of IHC were analyzed by photography under microscopy with 400× magnification and quantification with the Image-Pro^®^ Plus software.

### Cell culture

SW1353 cell line was obtained from American Tissue Culture Collection (ATCC), and chondrocyte cell line C28/I2 was kindly provided by Professor Junling Cao, from Institute of Endemic Diseases, Xi’an Jiaotong University Health Science Center. Both cell lines were cultured in RPMI-1640 medium (Hyclone, catalog SH30809.01) with 10% fetal bovine serum (FBS; ExCell, FCS500). Both cell lines were maintained at 37 °C and in the presence of 5% CO_2_ and cultured in monolayer and grown to confluence. The medium with 1% penicillin/streptomycin (Thermo Fisher, catalog 15140122) was changed every 2 days. When the cultures reached 90% confluence, the cells were detached by treatment with 0.05% trypsin (ExCell, CB000-C022) and passaged in culture.

### Target prediction and luciferase reporter assay

TargetScan (www.targetscan.org), miRDB (www.mirdb.org), and miRanda (www.microrna.org) were used to predict the target relationship between miR-23b-3p and *HS6ST2* mRNA. *HS6ST2* mRNA possesses a putative miR-23b-3p-binding site in its 3′UTR. Dual luciferase reporter assay was performed to validate the target relationship. The linker fragment (linker fragment primer sequences used for Dual-Luciferase vector are listed in supplementary Table S2) containing *HS6ST2* wild-type or mutant 3′UTR-binding site was synthesized and inserted into the pmirGLO Dual-Luciferase vector (Promega, catalog E133A). SW1353 cells were co-transfected with 100 ng of pmirGLO vectors possessing the wild-type or mutant 3′UTR of *HS6ST2* mRNA and 10 nM of mimic miR-23b-3p or 50 nM anti-miR-23b-3p with Lipofectamine 2000 (Thermo Fisher, catalog 11668-019) according to the manufacturer’s instructions. After 48 h, SW1353 cells were harvested and luciferase activity was assayed using the Dual Luciferase Reporter Assay System (Promega, catalog E1910).

### Chondrocyte treatment with TNF-α

SW1353 cells were seeded in 12-well plates at 7 × 10^4^ cells/well (C28/I2 seeded 1.2 × 10^5^ cells/well). After 24 h, the chondrocytes should be FBS free for >10 h, and then TNF-α (10 ng/ml, Sino Biological lnc, catalog HG10602-M) was used to stimulate cells for appropriate time (SW1353 for 24 h and C28/I2 for 6 h). In the experiment of miRNA and TNF-α double stimulation, the TNFα treatment was for 24 h (SW1353 cells) or 6 h (C28/I2 cells) after transfection and FBS free for at least 10 h.

### Transient transfection of mimic miR-23b or anti-miR-23b sequence

SW1353 cells were seeded in 12-well plates at 7 × 10^4^ cells/well (C-28/I2 at 1.2 × 10^5^ cells/well). After 24 h, 10 nM mimic miR-23b-3p (miR-23b) or negative control (NC) (Genepharma, Shanghai, China) and 50 nM of anti-miR-23b-3p sequence (anti-miR-23b) or negative control (anti-NC) (Genepharma, Shanghai, China) were transiently transfected into cells by Lipofectamine 2000 (Thermo Fisher, catalog 11668-019, 1.5 μl/well) according to the manufacturer’s instructions, respectively. Total RNA was isolated by using TRIzol Reagent (Thermo Fisher, catalog 15596-026), and whole-cell lysate was extracted with lysis buffer for western blotting assay after 48 h.

### Transient transfection of siRNA and plasmids

For the knockdown of HS6ST2 or p38 MAPK expression, siRNA against HS6ST2 (si-*HS6ST2*), p38 MAPK (si-*p38*), and the negative control siRNA (si-NC) were prepared (Genepharma, Shanghai, China). SW1353 cells were seeded in 12-well plates at 7 × 10^4^ cells/well. After 24 h, 50 nM siRNA mixture (containing three different target sequence) or si-NC were transiently transfected into the cells by Lipofectamine 2000 (Thermo Fisher, catalog 11668-019, 1.5 μl/well) and the efficiency of knockdown was determined by western blotting after transfection for 48 h. Different siRNA sequences against *HS6ST2* or *p38 MAPK* are listed in Supplementary Table S1.

Human *HS6ST2* CDS was cloned from chondrocyte cDNA of C28/I2 chondrocytes (primer sequences used for *HS6ST2* CDS clone are listed in Supplementary Table S2) and was inserted into pcDNA3.1 vector (Thermo Fisher, catalog VPI0001) with FLAG tag in the N-terminal. SW1353 cells seeded in 6-well plates at 2 × 10^5^ for 24 h, and pcDNA3.1-FLAG-*HS6ST2* vector (2 μg/well) or pcDNA3.1-FLAG empty vector (2 μg/well) were transiently transfected into cells by Lipofectamine 2000 (Thermo Fisher, catalog 11668-019, 3 μl/well). The expression of exogenous HS6ST2 was determined by anti-FLAG antibody (1:1000, Sigma, catalog F1804) in western blotting.

### Toluidine blue staining

SW1353 cells were seeded in 24-well plates at 3 × 10^4^ for 24 h. After transfection of miRNAs or plasmids, the chondrocytes were incubated with TNF-α (10 ng/ml, Sino Biological lnc, catalog HG10602-M) for another 24 h. In order to determine the matrix content, the chondrocytes were fixed in 4% buffered paraformaldehyde for at least 20 min and stained with 1% toluidine blue for 10 min (miRNA or siRNA transfection) or 15 min (plasmid transfection). After washing with PBS buffer, the results of staining were analyzed by photography under microscope with 200× magnification and quantification with the Image-Pro^®^ Plus software.

### Administration of p38 inhibitor to human cartilage cells

SW1353 cells were seeded in 12-well plates at 7 × 10^4^ for 24 h (C28/I2 1.2 × 10^5^ cells/well). After transfection with 10 nM mimic miR-23b-3p or 50 nM si-*HS6ST2* mixture for 24 h, the chondrocytes were incubated with or without SB203580 (10 μM, p38 MAPK chemical inhibitor, Selleck, catalog S1076). After 2 h, the cells were treated with TNF-α (10 ng/ml, Sino Biological lnc, catalog HG10602-M) for another 24 h (SW1353 cells) or 6 h (C28/I2 cells), and then the cells were harvested for further analysis.

### Reverse transcriptase-qPCR analysis in cells

Total RNA was extracted from the cells using the Trizol Reagent (Thermo Fisher, catalog 15596-026). cDNA was synthesized from 2 μg of total RNA in 20 μl of reaction volume using the Transcriptor cDNA Synthesize Kit (Roche, catalog 04897030001) with OligdT primer, and to detect the miR-23b-3p level the same cDNA sample was synthesized from 500 ng of total RNA with miR-23b-3p-specific stem-loop reverse transcription primer. Gene expression levels were measured by qPCR using the SYBR Green System (Roche, catalog 04913850001). Quantification of the relative expression levels was determined by ΔΔCt method. Triplicates were used in assay, and the cell experiments were repeated three times. All the primer sequences for mRNA detection are shown in Supplementary Table S2.

### Protein sample preparation and western blotting

Whole-cell lysate was prepared in western blotting and immunoprecipitation lysis buffer (Beyotime, catalog P0013B) containing protease inhibitor mixture (Bimake, catalog B14002) and phosphatase inhibitor mixture (Biomake, catalog B15002). The cell lysate were incubated in ice for 30 min and then centrifuged at 12,000 × *g* at 4 °C for 15 min, and the concentration of protein in cleared lysate was determined by BCA Protein Assay (TIANGEN, catalog PA115). Protein solution (about 15 μg) was first separated in 10% sodium dodecyl sulfate-polyacrylamide gel electrophoresis and then transferred to the polyvinylidene difluoride (PVDF) membrane (Millipore, catalog IPVH00010). The PVDF membrane with the protein should been blocked in 5% no fat milk or 5% BSA at room temperature for 2 h, and then the membranes were incubated with primary antibodies, including anti-HS6ST2 (1:400, Abcam, catalog ab122220), anti-MMP13 (1:500, R&D, catalog AF511), anti-MMP3 (1:1000, Abcam, catalog ab53015), anti-ADAMTS4 (1:1000, R&D, catalog AF4307), anti-ACAN (1:250, Millipore, catalog AB1031), anti-Phospho-JNK MAPK (1:1000, CST, catalog 9251), anti-JNK MAPK (1:1000, CST, catalog 9252), anti-Phospho-p38 MAPK(1:1000, CST, catalog 9211 s), anti-p38 MAPK (1:1000, CST, catalog 9212 s), anti-IκB (1:1000, Proteintech, catalog 10268-1-AP), anti-GAPDH (1:2000, Proteintech, catalog 60004-1-Ig), anti-FLAG tag (1:1000, Sigma, catalog F1804), anti-MEK3/6 (1:1000, Abcam, catalog ab200831), and anti-DUSP10 (1:1000, Abcam, catalog ab140123). All the primary antibodies were diluted with 5% BSA/TBST buffer (with 0.1% Tween 20) and were incubated overnight at 4 °C. After washing with TBST buffer (with 0.1% Tween 20) for three times, the membranes were incubated for 2 h with secondary horseradish peroxidase-conjugated anti-Mouse (1:10,000, Thermo Fisher, catalog A21202), anti-Rabbit (1:10,000, Thermo Fisher, catalog A11036), or anti-Goat IgG (1:10,000, Santa Cruz, catalog sc-2020). After washing with TBST and TBS, the target proteins were detected on membrane by ECL detection system (Millipore, catalog MA01821). Western blotting were scanned by GeneGnome XRQ System with the GeneTools analysis software (Syngene, MD, USA), and densitometry of specific western blotting bands was analyzed with the GeneTools analysis software.

### Statistics

All of the cell experiments were performed with at least three independent biological replicates. Bars represent standard error of the mean (SEM) from three independent experiments. Mann–Whitney *U* test was used to identify statistically differences between groups. Paired *t* test was used to identify differences between OA smooth cartilage and OA-damaged cartilage from the same patients. The correlation analysis was performed by using Spearman correlation. *P* value <0.05 was considered statistically significant.

## Electronic supplementary material

Supplementary Material

Supplementary Figure S1

Supplementary Figure S2

Supplementary Figure S3

Supplementary Figure S4

Supplementary Figure S5

Supplementary Figure S6

Supplementary Figure S7

Supplementary Figure S8
