# Supplementary material for: Downregulation of HS6ST2 by miR-23b-3p enhances matrix degradation through p38 MAPK pathway in osteoarthritis
Source: Cell Death Dis. 2018 Jun 13;9(6):699. doi: 10.1038/s41419-018-0729-0 (PMC5999974; doi:10.1038/s41419-018-0729-0)
Supplement: Supplementary file 1 — Supplementary Material [file 41419_2018_729_MOESM1_ESM.docx]

**Supplementary methods**

**Immunoprecipitation**

Proteins were immunoprecipitated from whole-cell lysates of SW1353 cells after transfection of pcDNA3.1-FLAG-*HS6ST2* vector or empty vector with or without 10 ng/ml TNF-α for 24h. SW1353 cells were incubated on ice for 30 min in Western and IP lysis buffer containing protease inhibitor mixture (Biomake) and phosphatase inhibitor mixture (Biomake). After centrifuged at 12,000 × g at 4 °C for 15 min, proteins in clear lysates were immunoprecipitated by incubating at 4 °C overnight with anti-FLAG tag Magnetic Beads (Biomake). After washing, proteins in immunoprecipitation complexes were eluted in SDS-reduction loading buffer and used for further Western blotting analysis.

**Supplementary Figure legends**

**Supplementary Figure S1. Downregulation of HS6ST2 at protein level in OA damaged cartilage tissues relative to smooth cartilage tissues.**

Expression of HS6ST2 protein was detected using immunohistochemical analysis between OA damaged cartilage and matched smooth cartilage from 16 pairs of OA patients. Scale bar: 100 μm.

**Supplementary Figure S2. Bioinformatics results between *HS6ST2* mRNA and miR-23b-3p**

**(a)** Schematic sequence match between miR-23b-3p and 3'UTR of *HS6ST2* gene. HS6ST2 gene was predicted as a target gene of miR-23b-3p with TargetScan and miRDB and *HS6ST2* mRNA 3'UTRs of humans, mice and rats were well matched with miR-23b-3p. **(b)** Diagrammatic structure of *HS6ST2* mRNA and target sequence location of miR-23b-3p in *HS6ST2* mRNA 3'UTR. **(c)** Schematic diagram of binding relationship between miR-23b-3p and wild-type or mutated *HS6ST2* mRNA 3'UTR.

**Supplementary Figure S3. Upregulation of miR-23b-3p and downregulation of HS6ST2 under treatment of TNF-α in C28/I2 chondrocyte.**

C28/I2 cells were stimulated by 10 ng/ml TNF-α for 6 h. The expression of miR-23b-3p was determined by stem-loop RT-qPCR (left panel), and the protein level of MMP13 and HS6ST2 were assayed by Western blotting (right panel). U6 snRNA and GAPDH were used as internal controls in RT-qPCR for miRNA and Western Blotting detection, respectively. Bars represent standard error of the mean (SEM) from three independent experiments. Here are one representative result and quantitative data from three independent Western blotting. Mann-Whitney U test was used to identify statistical differences between two groups. * stands for *P* value less than 0.05.

**Supplementary Figure S4. Upregulation of MMP13 and phosphorylation form of p38 MAPK under transfection of miR-23b-3p or siHS6ST2 in C28/I2 chondrocyte.**

**(a)** C28/I2 cells treated with 10 nM mimic of miR-23b-3p were stimulated by 10 ng/ml TNF-α for another 6 h, and protein level of HS6ST2, MMP13, p-p38, total p38 were determined by Western blotting. *: compared with mimic NC group. **(b)** C28/I2 cells treated with 50 nM si-*HS6ST2* were stimulated by 10 ng/ml TNF-α for another 6 h, and protein level of HS6ST2, MMP13, p-p38, total p38 were determined by Western blotting. *: compared with si-NC group. GAPDH was used as internal control. Each relative expression of phosphorylation form was normalized by using its total form. Here are one representative result and quantitative data from three independent Western blotting. Mann-Whitney U test was used to identify statistical differences between two groups. * or # stands for *P* value less than 0.05.

**Supplementary Figure S5. Detection of downstream signal pathway of TNF-α under stimulation of miR-23b-3p or anti-miR-23b-3p**

SW1353 cells treated with 10 nM mimic miR-23b-3p **(a)** or 50 nM anti-miR-23b-3p sequence **(b)** were stimulated by 10 ng/ml TNF-α for another 24 h, and the protein expression of p-p38, p38, p-JNK, JNK and IκB was detected by using Western blotting. GAPDH was used as internal control.

**Supplementary Figure S6. Inhibition of increasing *MMP13* mRNA level under treatment of SB203580.**

**(a)** SW1353 cells were stimulated by 10 ng/ml TNF-α with or without p38 MAPK inhibitor SB203580 (10 μM), and the *MMP13* mRNA expression was assayed by RT-qPCR. **(b, c)** SW1353 cells were treated with mimic miR-23b-3p (b) or si-*HS6ST2* (c) with or without inhibitor SB203580 (10 μM) under stimulation of TNF-α. The mRNA expression of *MMP13* was determined by RT-qPCR. *: compared with mimic NC group (b) or si-NC group (c). GAPDH was used as internal control. Mann-Whitney U test was used to identify statistical differences between two groups. * stands for *P* value less than 0.05.

**Supplementary Figure S7. Upregulation of MMP13 under transfection of miR-23b-3p or si-*HS6ST2* depending on activity of p38 MAPK in C28/I2 chondrocyte.**

**(a, b)** C28/I2 cells were transfected with 10 nM mimic miR-23b-3p (a) or 50nM si-*HS6ST2* (b) with or without p38 MAPK inhibitor SB203580 (10 μM) under treatment of TNF-α for 6 h, and the protein expression of MMP13 was determined by Western blotting. *: compared with mimic NC group (a) or si-NC group (b). Here are one representative result and quantitative data from three independent Western blotting and GAPDH was used as internal control. Mann-Whitney U test was used to identify statistical differences between two groups. * or # stands for *P* value less than 0.05. NS stands for not significant.

**Supplementary Figure S8. No interaction between HS6ST2 and MEK3/6, p38 MAPK and DUSP10.**

Whole-cell lysates from SW1353 cells transfected by pcDNA3.1-FLAG-*HS6ST2* vector(FLAG-*HS6ST2*) or empty (FLAG-Ctrl) with or without TNF-α for 24h were co-immunoprecipitated with anti-FLAG tag Magnetic Beads, and immunoblotted with anti-HS6ST2, anti-p38 MAPK, anti-Phospho-p38 MAPK, anti-MEK3/6 and anti-DUSP10 antibodies, respectively.

**Supplementary Tables**

**Supplementary Table S1. Sequences of siRNA oligonucleotides**

| Gene name | siRNA oligonucleotides sequences (5′-3′) |
| --- | --- |
| hsa-HS6ST2 siRNA1 sense | CUCUUCCUAUUUGCCGUGATT |
| hsa-HS6ST2 siRNA1 antisense | UCACGGCAAAUAGGAAGAGTT |
| hsa-HS6ST2 siRNA2 sense | CAUGAACUUUAUUUCGCCATT |
| hsa-HS6ST2 siRNA2 antisense | UGGCGAAAUAAAGUUCAUGTT |
| hsa-HS6ST2 siRNA3 sense | CGACUACAUAGGCAGUGUATT |
| hsa-HS6ST2 siRNA3 antisense | CGACUACAUAGGCAGUGUATT |
| hsa-p38 MAPK siRNA1 sense | GAGCUGAACAAGACAAUCUTT |
| hsa-p38 MAPK siRNA1 antisense | AGAUUGUCUUGUUCAGCUCTT |
| hsa-p38 MAPK siRNA2 sense | GUCCAUCAUUCAUGCGAAATT |
| hsa-p38 MAPK siRNA2 antisense | UUUCGCAUGAAUGAUGGACTT |
| hsa-p38 MAPK siRNA3 sense | GGUCUCUGGAGGAAUUCAATT |
| hsa-p38 MAPK siRNA3 antisense | UUGAAUUCCUCCAGAGACCTT |
| siRNA negative control sense | UUCUCCGAACGUGUCACGUTT |
| siRNA negative control antisense | ACGUGCCACGUUCGGAGAATT |

**Supplementary Table S2. Primers used for stem-loop reverse transcription, polymerase chain reaction of microRNAs and messenger RNA and CDS clone.**

| Gene name | Primer sequences (5′-3′) |
| --- | --- |
| hsa-miR-23b-3p stem-loop RT primer | GTCGTATCCAGTGCAGGGTCCGAGGTATTCGCACTGGATACGACGGTAAT |
| hsa-miR-23b-3p stem-loop forward | CGCATCACATTGCCAGGG |
| miRNA stem-loop reverse | GTGCAGGGTCCGAGGT |
| U6 forward | CTCGCTTCGGCAGCACA |
| U6 reverse | AACGCTTCACGAATTTGCGT |
| hsa-HS6ST2 forward | CCAAGTCAAATCTGAAGCACA |
| hsa-HS6ST2 reverse | TCTGGAAATGGGTCTGAAGGA |
| hsa-ACAN forward | ATGCCCAAGACTACCAGTGG |
| hsa-ACAN reverse | TCCTGGAAGCTCTTCTCAGT |
| hsa-MMP13 forward | AATATCTGAACTGGGTCTTCCAAAA |
| hsa-MMP13 reverse | CAGACCTGGTTTCCTGAGAACAG |
| hsa-MMP3 forward | CTGGACTCCGACACTCTGGA |
| hsa-MMP3 reverse | CAGGAAAGGTTCTGAAGTGACC |
| hsa-ADAMTS4 forward | GGTCAAGGTCCCATGTGCAAC |
| hsa-ADAMTS4 reverse | GAATGCGGCCATCTTGTCATC |
| hsa-HS6ST2 miR-23b 3'UTR-wt olig forward | CTAGCGGCCGCTTAACCTAAAATGTGAACTCTTACTTT |
| hsa-HS6ST2 miR-23b 3'UTR-wt olig reverse | CTAGAAAGTAAGAGTTCACATTTTAGGTTAAGCGGCCGCTAGAGCT |
| hsa-HS6ST2 miR-23b 3'UTR-mt olig forward | CTAGCGGCCGCTTAACCTAACCATGCTACTCTTACTTT |
| hsa-HS6ST2 miR-23b 3'UTR-mt olig reverse | CTAGAAAGTAAGAGTAGCATGGTTAGGTTAAGCGGCCGCTAGAGCT |
| hsa-HS6ST2 CDS clone forward | CCCAAGCTTATGGCACTGCCTGCGTGT |
| hsa-HS6ST2 CDS clone reverse | CGGGATCCTTAACGCCATTTCTCTACACTGC |

**Author contributions**

This project was conceived and supervised by J. S. and S.L. Most of the experiments were performed by Y.G., Z.M., C.J., W.W., J.Y., J.X., M.S., Y.Z. and S.H. The OA patients’ sample collection was contributed by P.X., K.X., Y.H. and F.Z. The manuscript was prepared by Y.G., W.Z., D.L., L.M., J.S. and S.L.

In Figure 1, P.X. and K.X. collected the articular cartilage from OA patients. Y.G., Z.M. and C.J. generated the RT-qPCR data from articular cartilage, and Y.G., Z.M. ,Y.H. and F.Z generated the histology and immunohistochemistry data and labelled the image. Y.G. generated the correlation analysis. J.S. and S.L. assembled the figures.

In Figure 2, W.W. and J.Y. generated target prediction and luciferase reporter assay. Y.G. and C.J. generated the RT-qPCR data. Y.G., Z.M. and M.S. generated the Western Blotting data. Y.Z. and S.H assembled the figures.

In Figure 3, Y.G., Z.M., C.J., W.W. generated the Western Blotting data of cell samples under TNF-α stimulation. J.Y. and J.X. were in charge of miRNA transfection. C.J. and Z.M. generated the RT-qPCR data. Y.G. generated the toluidine blue staining results. J.S. and S.L. assembled the figures.

In Figure 4, Y.G. was in charge of vector construction. J.Y. and J.X. were in charge of siRNA and plasmids transfection. Y.G., Z.M., C.J. generated the Western Blotting data. Y.G. generated the toluidine blue staining results. Y.Z. and S.H assembled the figures.

In Figure 5, J.Y. and J.X. were in charge of siRNA and plasmids transfection and treatment of inhibitor. Y.G. and C.J. generated the Western Blotting data. Y.Z. and Z.M. assembled the figures.

In Figure 6, Y.G. and C.J. were in charge of siRNA and plasmids transfection. Y.G. and Z.M. generated the Western Blotting data. Y.G. and M.S. generated the mechanism diagram. J.S. and S.L. assembled the figures.
