# Supplementary figures and images for: Downregulation of HS6ST2 by miR-23b-3p enhances matrix degradation through p38 MAPK pathway in osteoarthritis
Source: Cell Death Dis. 2018 Jun 13;9(6):699. doi: 10.1038/s41419-018-0729-0 (PMC5999974; doi:10.1038/s41419-018-0729-0)

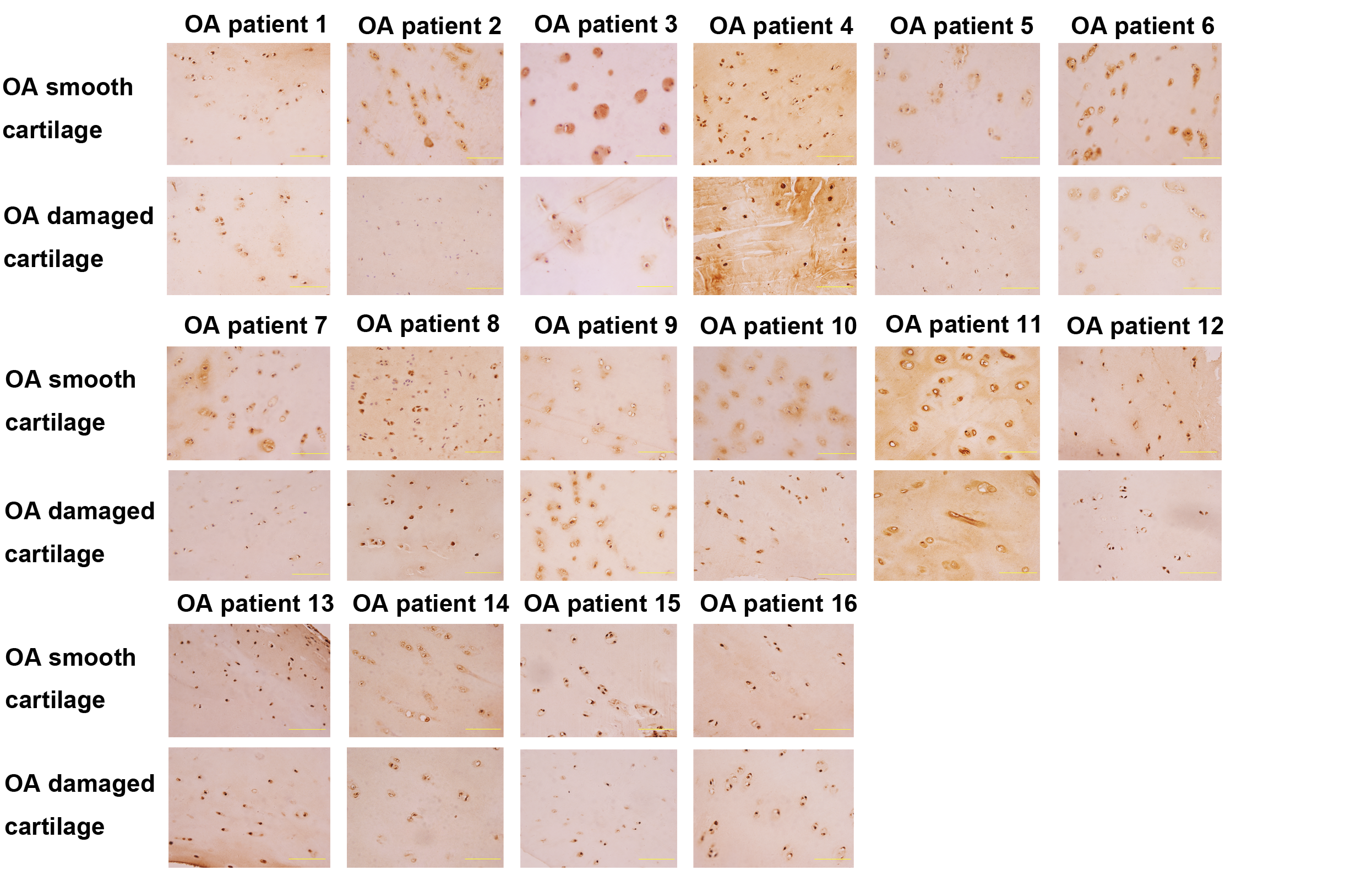

Supplement: Supplementary file 2 — Supplementary Figure S1 [file 41419_2018_729_MOESM2_ESM.tif]

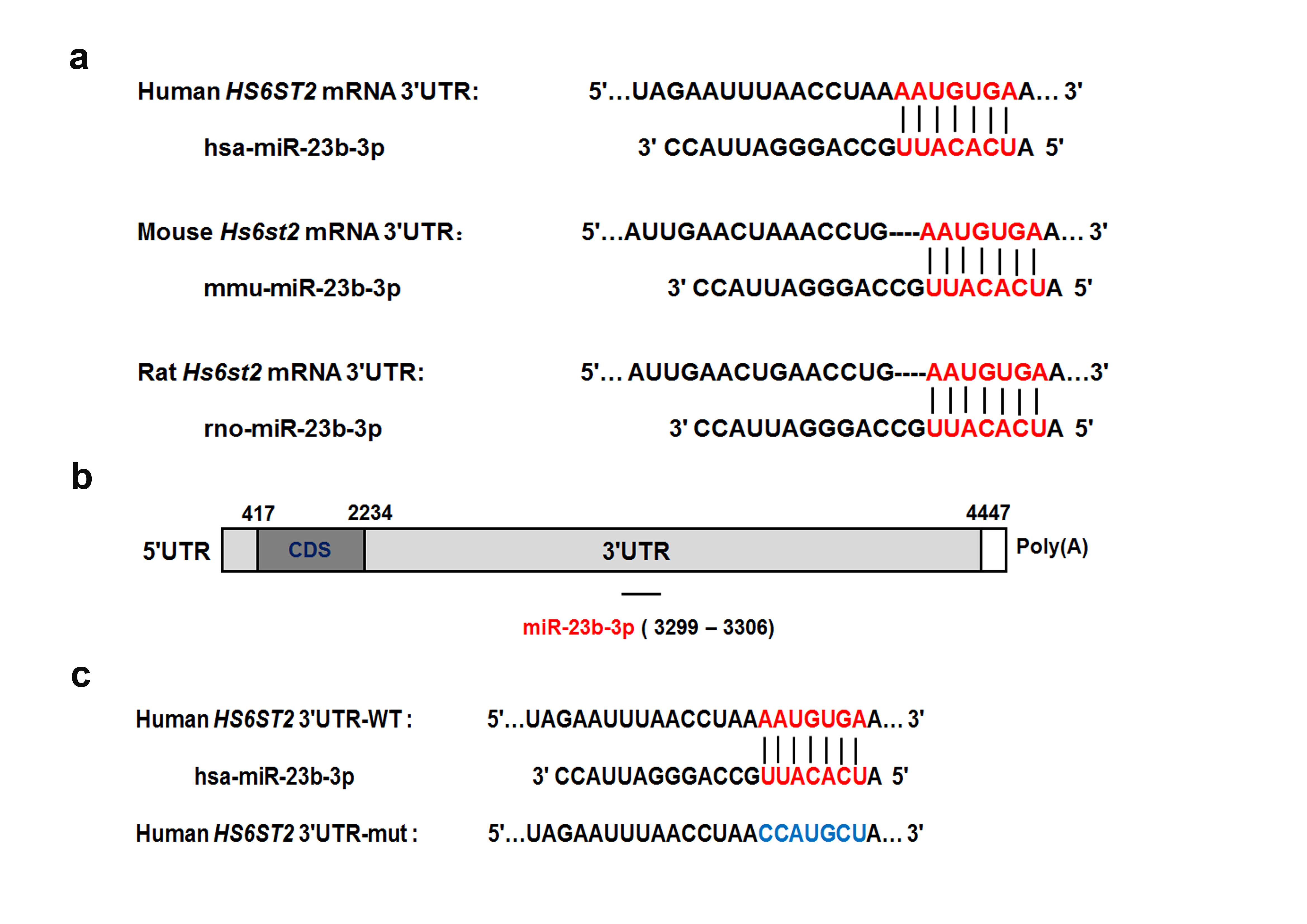

Supplement: Supplementary file 3 — Supplementary Figure S2 [file 41419_2018_729_MOESM3_ESM.tif]

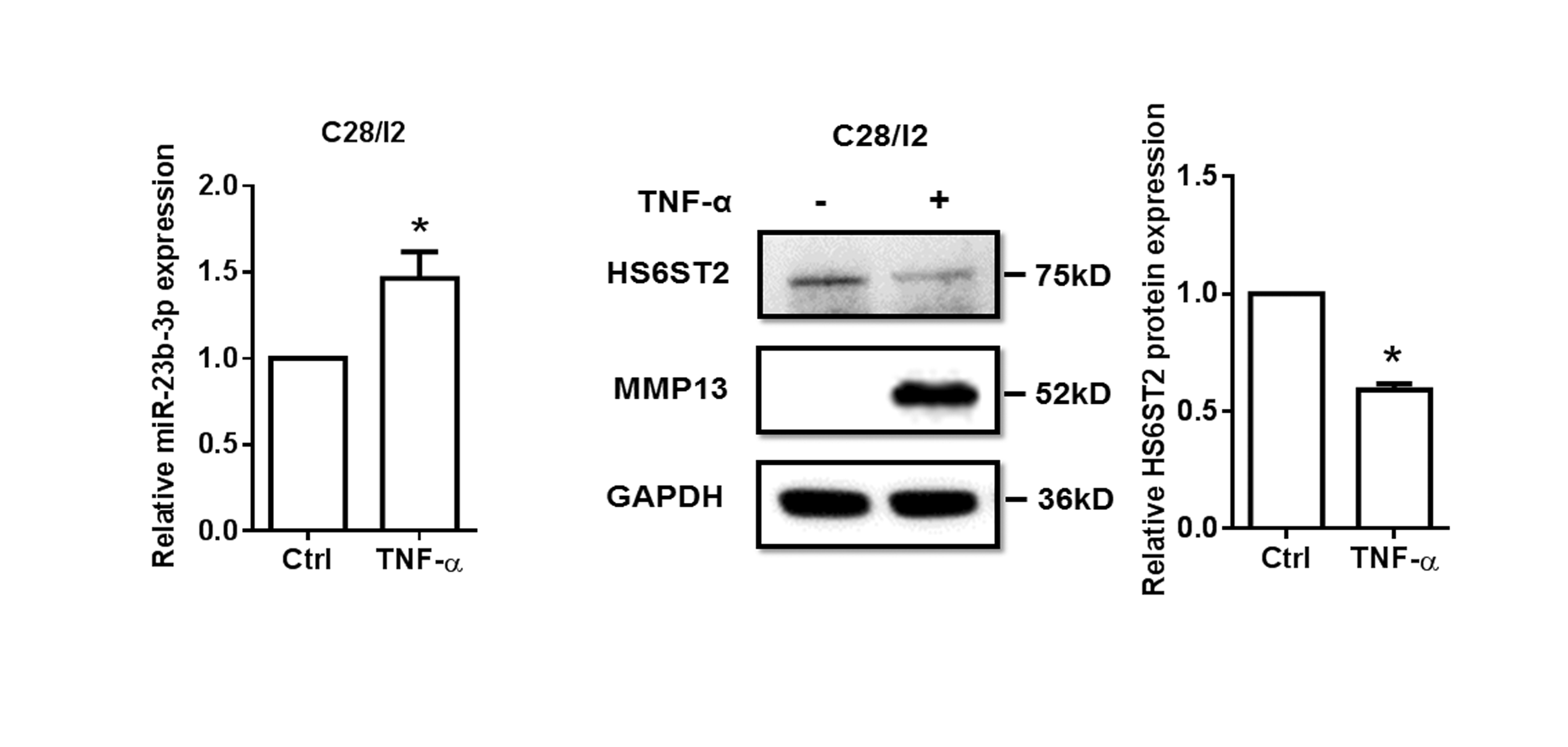

Supplement: Supplementary file 4 — Supplementary Figure S3 [file 41419_2018_729_MOESM4_ESM.tif]

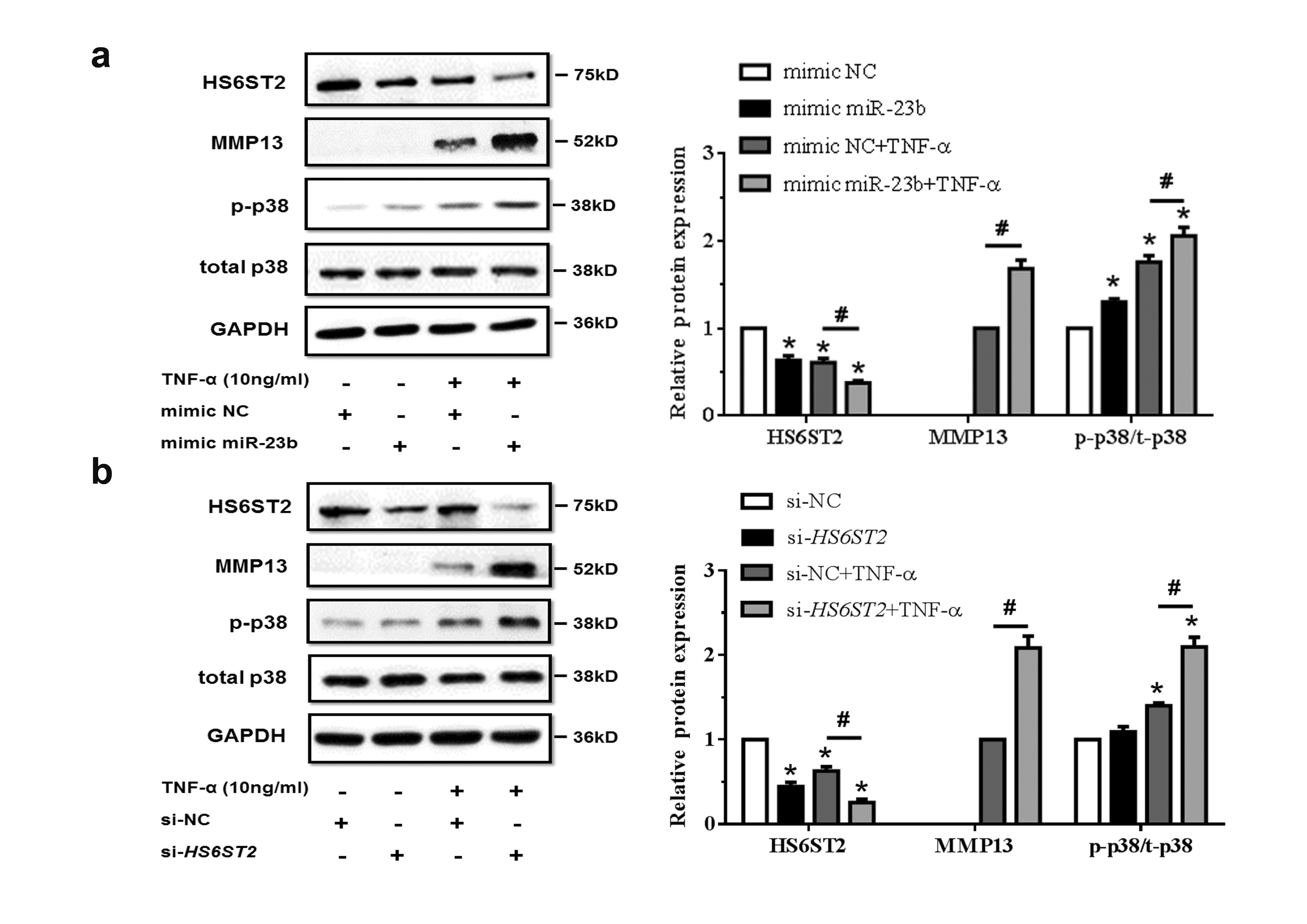

Supplement: Supplementary file 5 — Supplementary Figure S4 [file 41419_2018_729_MOESM5_ESM.tif]

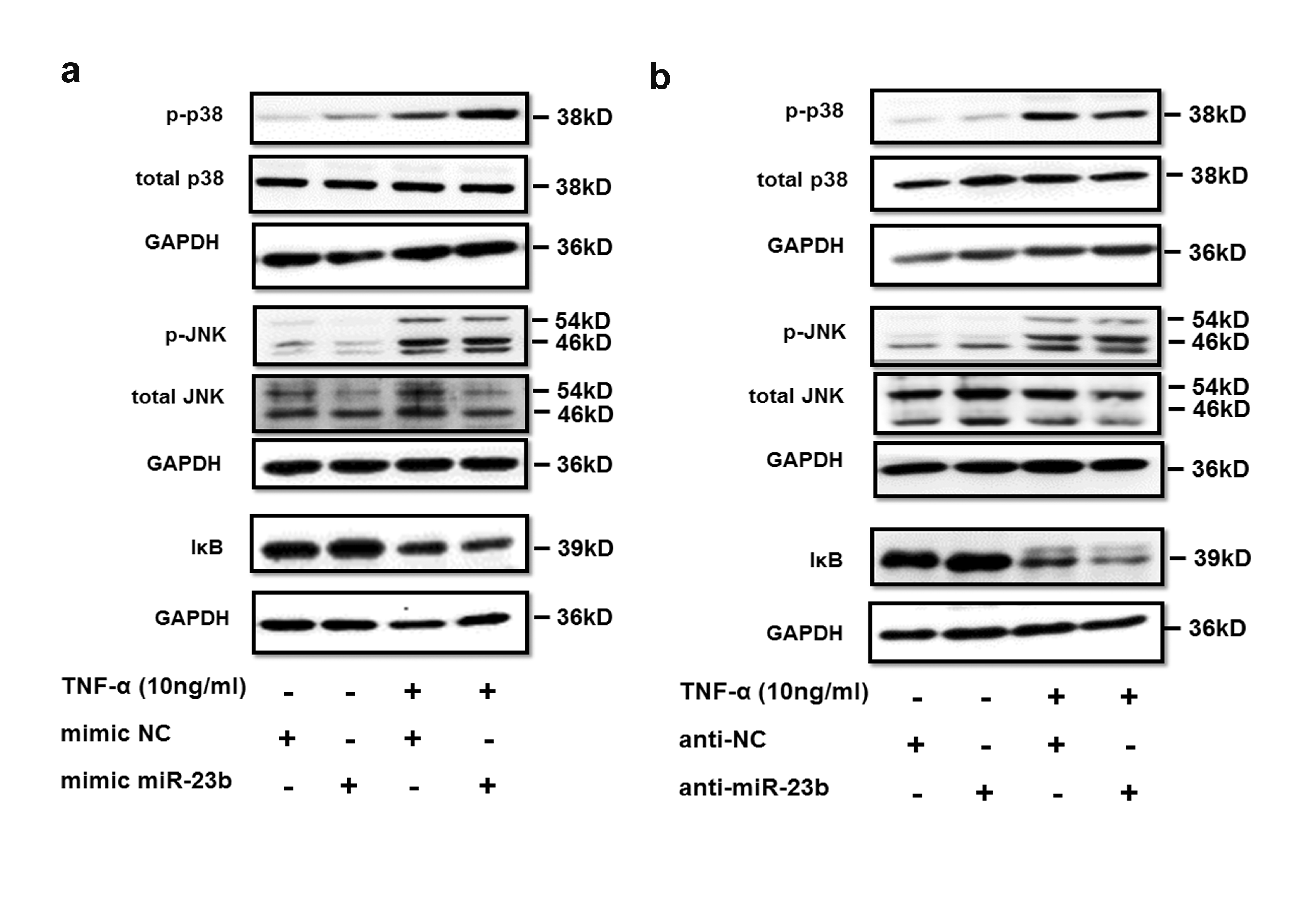

Supplement: Supplementary file 6 — Supplementary Figure S5 [file 41419_2018_729_MOESM6_ESM.tif]

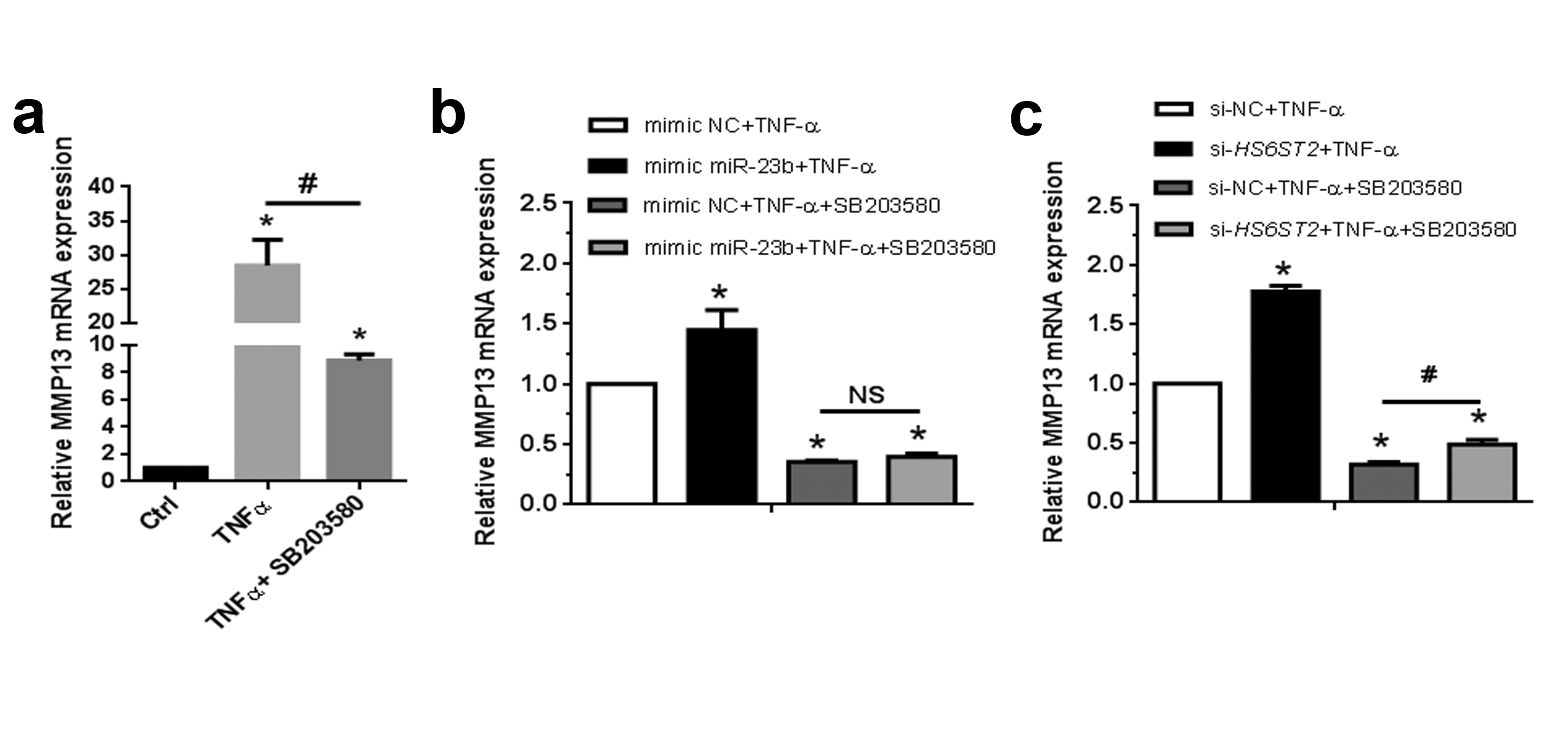

Supplement: Supplementary file 7 — Supplementary Figure S6 [file 41419_2018_729_MOESM7_ESM.tif]

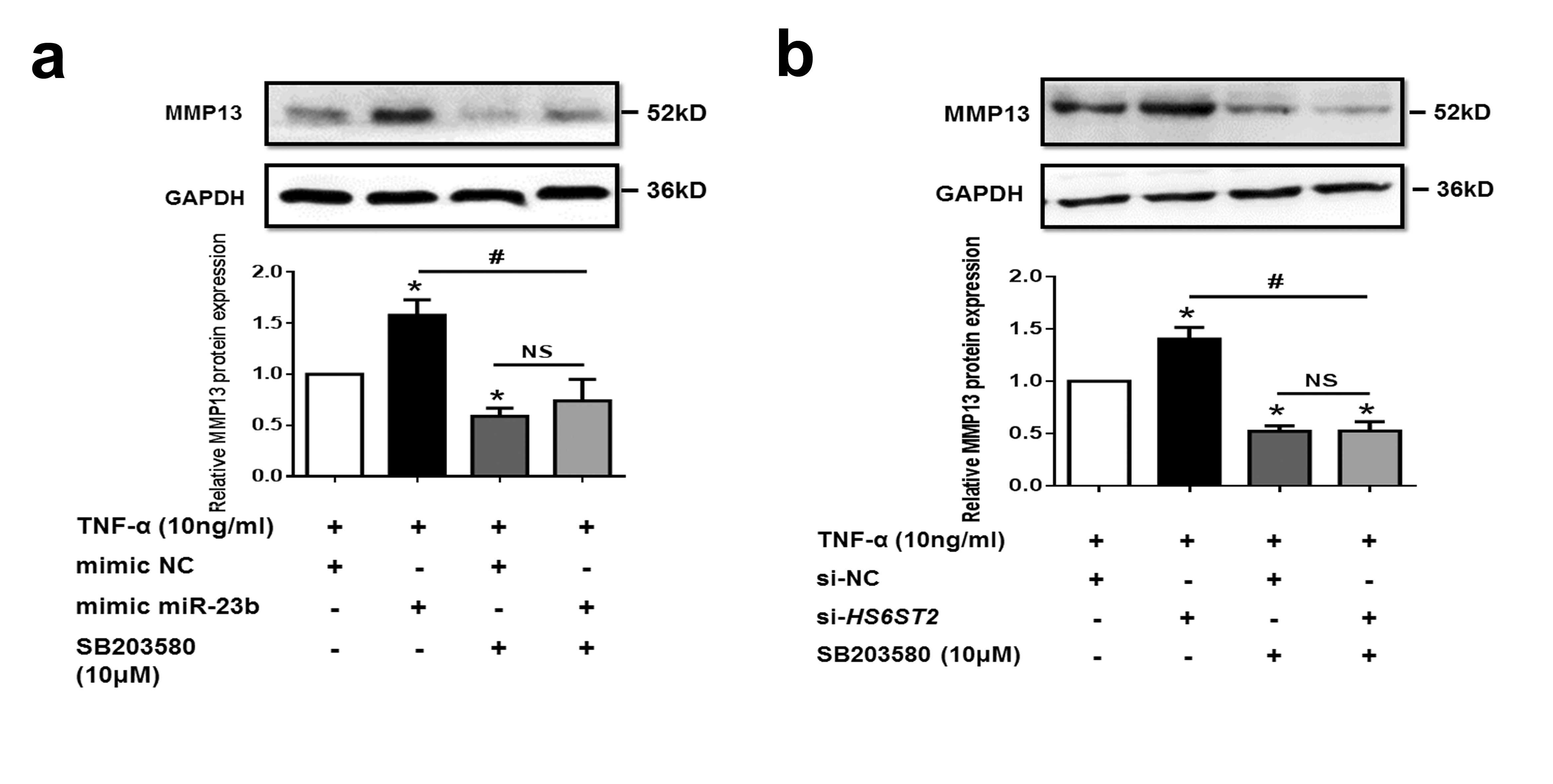

Supplement: Supplementary file 8 — Supplementary Figure S7 [file 41419_2018_729_MOESM8_ESM.tif]

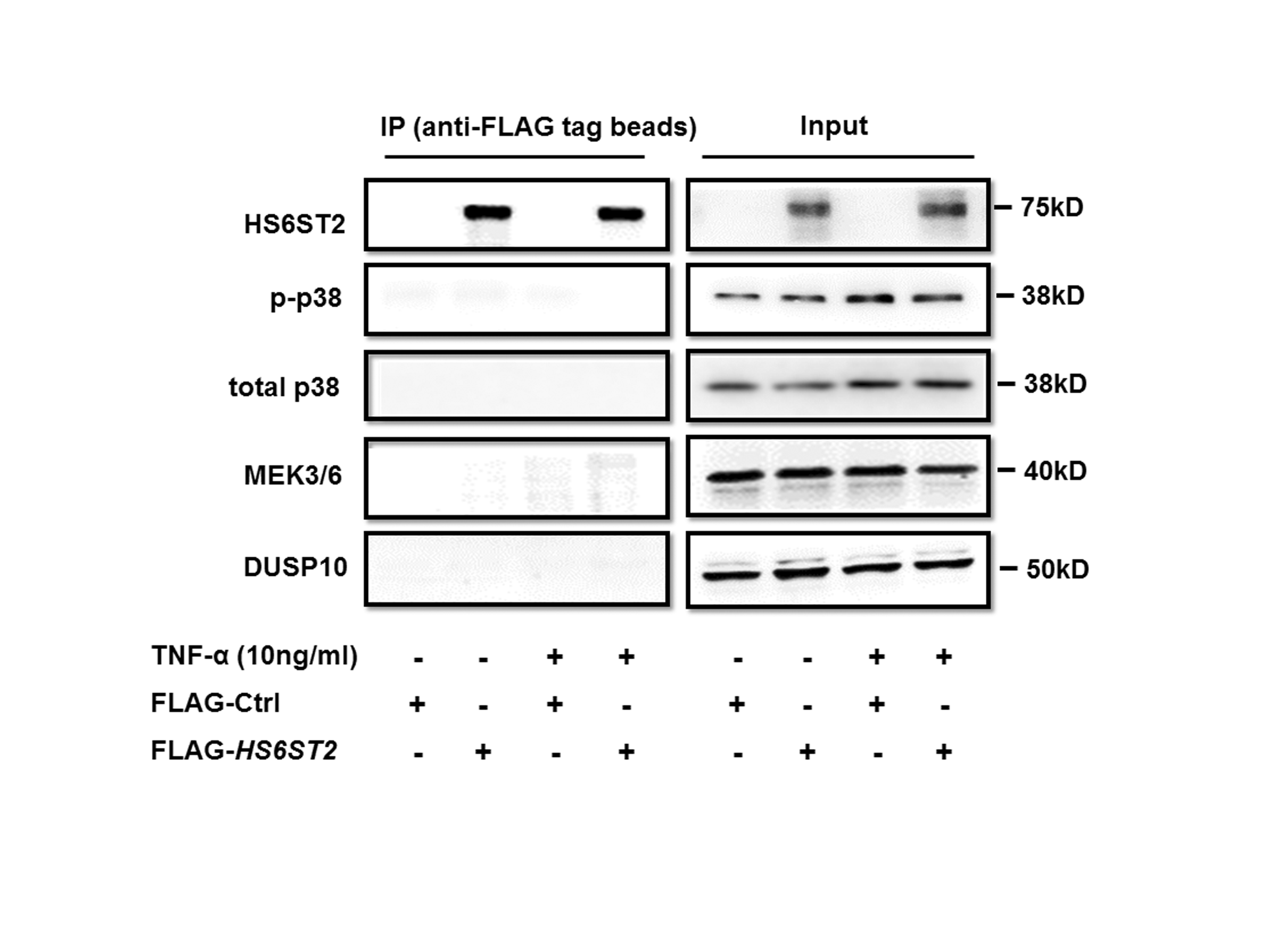

Supplement: Supplementary file 9 — Supplementary Figure S8 [file 41419_2018_729_MOESM9_ESM.tif]
